# Robust corrosion guard, mechanical and UV aging properties of metal complex/epoxy hybrid composite coating for C-steel applications

**DOI:** 10.1038/s41598-022-16348-3

**Published:** 2022-07-21

**Authors:** A. M. Fadl, S. A. Sadeek, Laila Magdy, M. I. Abdou

**Affiliations:** 1 Production Department, Egyptian Petroleum Research Institute, Nasr City, Cairo Egypt; 2 Department of Chemistry, Faculty of Science, Zagazig University, Zagazig, 44519 Egypt

**Keywords:** Engineering, Materials science

## Abstract

Incorporation of novel-prepared metal–organic complexes as crosslinking accelerators for multifunctional epoxy was on top of interest by coating formulators. The present work investigated the loading of mixed ligand metal complexes (Zr(IV) and Cu(II)) of metformin (MF) and 2.2′bipyridine (Bipy) against the free ligands as crosslinking modifiers via some epoxy coating formulations to assess their superb performances on the C-steel surface. Zr(IV) and Cu(II) demonstrated the minor energy gap (∆E) values at 0.190 au compared to free MF and Bipy according to the calculated energy values, and this behavior reflected their enhanced properties via epoxy coating applications. EIS measurements using high saline formation water as a corrosive medium were performed and offered that PA-DGEBA/MC-Cu coated film showed the superior resistance values (R_ct_ = 940 and R_c_ = 930 kΩ cm^2^). The accelerated corrosion salt spray experiment clarified that PA-DGEBA/MC-Cu coating achieved the least corrosion rate at 0.00049 mm/y and exhibited the highest protection efficiency of 99.84%. SEM/EDX combination survey affirmed the protective performance of the checked coatings. AFM microanalysis confirmed that surface-treated Cu(II) coating displayed the smoothest film surface with complete curing. Mechanical durability properties were evaluated and the obtained results illustrated that pull-off adhesion for PA-DGEBA/MC-Cu coated film fulfilled the highest adhesion strength at 6.3 MPa, the best bend character at 77, and the maximum impact resistance at 59.7 J. UV immovability trial was performed at 10 irradiance and 80 h duration. PA-DGEBA/MC-Cu coated film displayed the highest resistance to UV irradiance with blistering (#8 size and few frequencies) in addition to offering a minor gloss variation and matt properties.

## Introduction

Epoxy coatings (ECs) displayed excellent anti-corrosion, binding, chemical, and mechanical properties and demonstrated super-hydrophobic characteristics with efficiently curing applied on different steel structures^1–6^. At room temperature, epoxy vehicle displayed robust adhesion and low curing shrinkage after application on the metal surface^7^. The addition of crosslinking agents and anti-corrosion filling materials to epoxy coatings supported the barrier characteristics, in which prohibited the transportation paths of aggressive species via the coating layer^8–10^. Modifications of polymeric epoxy coatings were in progress to improve their protective ability^10–12^. Metformin (MF) formed Schiff base compounds by the reaction with aldehyde derivatives and offered coordinative and biological characteristics in addition to creating complexes by reacting with some metal ions^13–16^. Large scale applications used 2,2^\^-Bipyridine (Bipy) hetero ligand in macromolecular and supramolecular reactions^17,18^. As a polycyclic aromatic ligand, Bipy offered superb photo-optical, electrochemical, and photo-physical properties in addition to reacting with metal ions via N-donor heterocyclic atoms to form active metal complexes^19–23^.

Some multifunctional Schiff base metal complexes were synthesized and evaluated via epoxy hybrid nanocomposites as super crosslinkers for supporting the coating curing on the C-steel surface^24^. The results revealed the outstanding corrosion-inhibiting behavior and mechanical durability properties of paramagnetic complexes dispersed through coating layers due to their high electronegativity, enhancement in the crosslinking intensity and reinforcing the steel chemical bonding and film adhesion strength^25^. Some transition metal complexes were prepared to offer fire-resistant, dielectric, and wear resistance characteristics during the well dispersion through epoxy coating^26^. The fabricated Mn(II) complexes were used as driers alternative to Co(III) compounds for the alkyd coating layer^27^. Some fabricated and identified aluminum complexes were applied as driers (super crosslinkers) for alkyd resins and offered multifunctional characteristics^28^.

This work related with novel applications and discussions based on illustrating the relation between magnetic moment, electronegativity, electronic transition, and energy gap evaluations of mixed Zr(IV) and Cu(II) complexes of MF and Bipy ligands and their corrosion inhibiting, mechanical, and UV durability characteristics through the incorporation with an epoxy coating applied on the steel surface. Illustrations for electronegativity and energy gap values for the investigated compounds were made using DFT measurements and measuring their electronic transitions using electronic transition configurations. With the modification of epoxy binder with the same concentrations of the employed modifiers (ligands and their complexes), the coating's corrosion rate, protection efficiency, mechanical and UV durability characteristics were evaluated according to the ASTM standard methods for asserting their efficiency during application.

## Experimental work

### Materials and instruments

**Figure 1:**
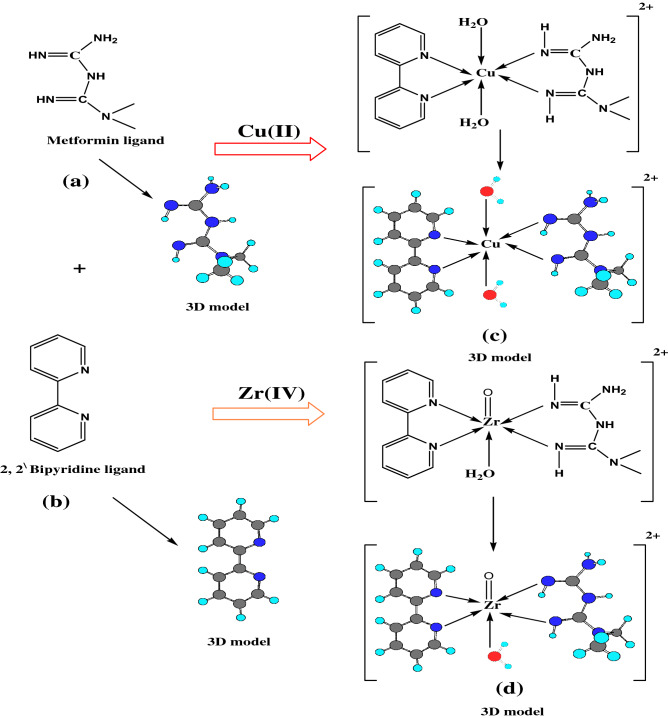
Molecular structures of (**a**) MF, (**b**) Bipy, (**c**) Cu(II) and (**d**) Zr(IV) and their 3D models.

MF, Bipy, as offered in Fig. 1a and b, and acetone were obtained from BDH, Sigma Aldrich. The prepared solid complex with dark blue color [Cu(M.F.)(Bipy)(H_2_O)_2_]Cl_2_.2H_2_O and the solid pink one [ZrO(M.F.)(Bipy)H_2_O]Cl_2_ were obtained from the chemistry department, Zagazig University. Their physical-analytical data and elemental analysis, in addition to the UV. Vis. Electronic transitions were offered in Tables 1 and 2^29^. Furthermore, the given energy values of investigated compounds were delivered^29^ and depicted in Table 3. The employed hardener (Ancamine 1734) with the chemical composition based on 4,4'-diamino diphenylmethane was gained from Anchor Chemicals. Epoxy binder (BECKOPOX™ EP 128, Solvent-free) was obtained from Coating Allnex Company, Germany. The utilized organic solvents and DOP plasticizer were delivered from El-Mohandes Company for Chemicals, Egypt. DMSO and THF were used with high purification and acquired by Sigma Aldrich Company.‏ C-Steel (CS) specimens used to study the investigated electrochemical behavior of coatings were divided with proportions of 10 × 10 × 0.8 mm. CS sheets were provided with 150 × 100 × 2 mm proportions for the accelerated corrosion cabinet (salt spray test). CS coupons employed for evaluating the mechanical and UV resistance properties of coatings were with dimensions of 150 × 100 × 0.8 mm. Sandblasting for mechanical surface preparation was done to achieve a surface roughness of 50 µm. Then, chemical treatment was performed to remove the surface contaminations by using distilled water and acetone for perfect coating application. The C-steel coupons was chemically analyzed as follows; Mn: 1.440%, Al: 0.023%, Cr: 0.590%, C: 0.090%, P: 0.190%, Ni: 0.220%, Cu: 0.150%, Si: 0.437%, V + Ti: 0.020%, Mo: 0.050%, and the Balance was Fe. The utilized test solution was the produced formation water submitted from Qarun Petroleum Company (QPC), Egypt. The chemical analysis of produced water was illustrated by ionic concentrations (ppm, w/w), as Na^+^ and K^+^: 53,165; Ca^2+^: 31,000; Mg^2+^: 2700; Cl^−^: 167,835; SO_4_^2−^: 350; HCO_3_^−^: 85 and the total TDS was 221,000. The pH of this produced water was 6.23, specific gravity; 1.109, salinity as NaCl; 192,755 ppm, wt, and the dissolved oxygen concentration; was 0.3 ppm, wt. The aggressiveness of used formation water solution could be mainly described due to the followings: (i) Formation water was containing on high ratios of dissolved gases such as hydrogen sulfide (H_2_S, which formed with C-steel, the sour corrosion products) and carbon dioxide (CO_2_, which was responsible for sweet corrosion). Oxygen was frequently found in the produced water, which also participated in the aggressiveness of this water, but it had little ratio and effect.

**Table 1:** Elemental analysis and physico-analytical data for MF, Bipy and their metal complexes.

Compounds M.Wt. (MF.)	Color (yield %)	M.P. (°C)	(calcd) % found				Λ S cm^2^ mol^-1^	μ_eff (B.M)_
			C	H	N	Cl		
MF 165.50(C_4_H_12_N_5_Cl)	White	224	(29.00) 28.90	(7.25) 7.18	(42.30) 42.15	(21.45) 21.35	55.80	–
Bipy 156(C_10_H_8_N_2_)	White -	72	(76.89) 76.81	(5.16) 5.11	(17.93) 17.91	**–**	9.56	–
Cu(II) 491.55(C_14_H_27_N_7_O_4_Cl_2_Cu)	Dark blue 93	230	(34.18) 34.11	(5.49) 5.41	(19.94) 19.90	(14.44) 14.37	121.12	1.70
Zr(IV) 481.22(C_14_H_21_N_7_O_2_Cl_2_Zr)	Pink 90	300	(34.91) 34.80	(4.36) 4.30	(20.36) 20.31	(14.75) 14.71	148.80	–

**Table 2:** The given UV–Vis. electronic transitions of MF, Bipy and their metal complexes.

Assignments (nm)	MF	Bipy	Cu(II)	Zr(IV)
π-π* transitions	290	284	295	295
n-π* transitions	310, 360, 373	347	320, 370	320, 370
Ligand–metal charge transfer	–	–	540	523
d-d transition	–	–	620	–

**Table 3:** Energy values (HOMO, LUMO, Energy gap ∆E/au, hardness (η), global softness (S), electronegativity (χ), absolute softness (σ), chemical potential (Pi), global electrophilicity (ω) and additional electronic charge (∆N_max_) of the two ligands and studied complexes by using DFT calculations.

Parameters	MF	Bipy	Cu(II)	Zr(IV)
HOMO, H	− 0.382	− 0.425	− 0.359	− 0.387
LUMO, L	− 0.017	− 0.145	− 0.169	− 0.197
I = − H	0.382	0.425	0.359	0.387
A = − L	0.017	0.145	0.199	0.197
∆E = L − H	0.365	0.280	0.190	0.190
η = (I − A)/2	0.183	0.140	0.095	0.095
χ = − (H − L/2)	0.199	0.285	0.294	0.292
σ = 1/η	5.465	7.143	10.526	10.526
S = 1/2 η	2.732	3.571	5.263	5.263
Pi = − χ	− 0.199	− 0.285	− 0.264	− 0.292
ω = (Pi)^2^/2 η	0.108	0.290	0.367	0.449
∆N_max_ = χ/η	1.087	2.036	2.779	3.074

(I) is ionization energy, (A) is an electron affinity.

 QUANTA FEG 250 equipment was used for SEM/EDX combination surface morphology investigation after exposing the surface-modified and conventional epoxy coatings to the severe saline salt spray atmosphere with a magnification of 800 X. 3D- images were taken utilizing Atomic force spectroscopy (AFM) analysis using C3000 system, Nanosurf Flex-Axiom to detect the surface coating microstructure, roughness and deteriorated zones with derived data fittings for the applied coatings. In addition, AFM analysis was applied to survey the effect of UV rays on the coated steel film after irradiance of 10 for 80 h duration.

### DFT theoretical investigations

Energy and atomic charge parameters using DFT theoretical survey for the modifier compounds was investigated using the B3LYP functional and Lee, Yang Parr’s, and Hartree–Fock local exchange function^30–34^.

### Coating design and formulation

**Figure 2:**
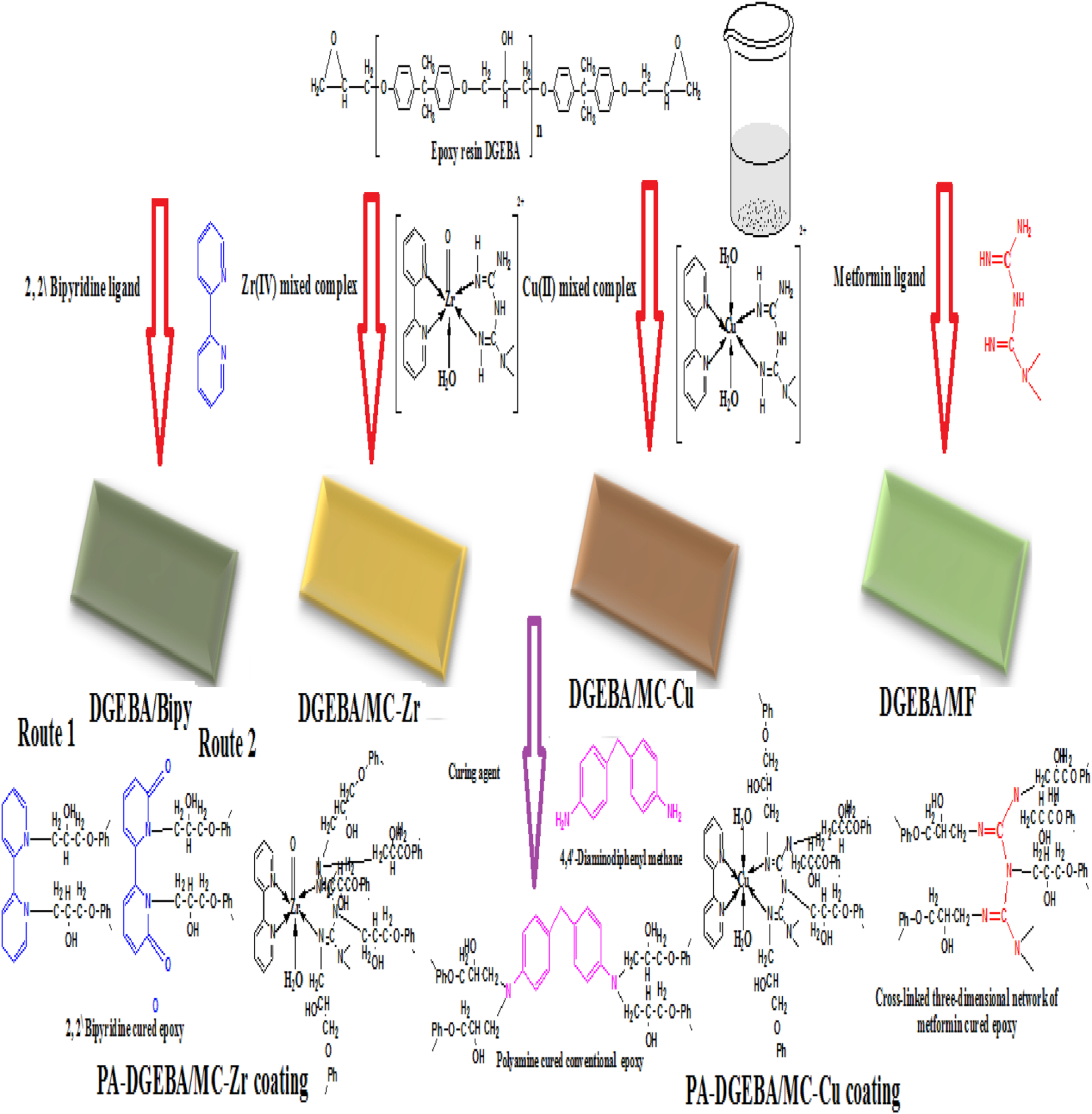
The suggested three-dimensional networks of cured epoxy with MF, Bipy ligands and their mixed Zr(IV), and Cu(II) complexes.

By using ultra-sonication, dissolve 0.1 g by weight of the employed modifiers (MF, Bipy, Zr(IV), and Cu(II)) in 50 ml of admixture solvent consisting in percent (%), by weight of 20 isobutyl alcohol: 50 DMSO: 30 THF. After that, incorporate 1% by weight of the dissolved modifier to the epoxy resin of DGEBA type. Then, isopropanol and xylene solvents were added with 0.2% ratio by weight of DOP plasticizer to construct the formulated hybrid modified coatings (DGEBA/MF, DGEBA/Bipy, DGEBA/MC-Zr, and DGEBA/MC-Cu), versus conventional epoxy (blank). The curing process was obtained using Ancamine 1734 and incorporated as (0.6 Hardener: 1 Epoxy resin) by ratios. The DFT of coatings was checked using the magnetic thickness gauge in the 70 ± 5 µm range. The illustrated crosslinking routes of epoxy resin with the modifier compounds were described in Fig. 2.

### The protective performance investigation

Electrochemical impedance spectroscopic (EIS) measurements were carried out using Voltalab 80 Potentiostate PGZ 402 with Voltamaster 4 software for measuring the electrochemical parameters. The employed frequency was used from 10 kHz to 100 MHz range. Three-electrode cell was utilized to estimate the electrochemical demeanor (coated steel pieces represented as the working electrode (WE), a saturated calomel electrode (SCE) as the reference electrode, and platinum wire as a counter electrode (CE). EIS was carried out at *E*_*ocp*_ (steady-state open circuit potential) by immersing the WE in the test solution for 90 min. The protective behavior analysis based on the weight loss method was implemented using the salt spray corrosive fog by calculating the corrosion rate (CR) and protection efficiency (PE) for the employed coatings. The steel coupons were prepared according to ASTM B117-03. 0.0001 g accuracy digital balance was utilized to measure the weight of coated specimens before and after performing the salt spray test (5% NaCl for 500 h), removing the formed rusting by utilizing 1 M HCl was carried out. Then after, washing with distilled water and dimethyl ketone in addition to complete drying was made by air. The protective behavior could be esteemed by measuring the CR, mm/y and PE % using the following Eqs. (1) and (2)^4,25^:

1 $${\text{CR}} = { }\frac{{\left( {\Delta {\text{W}}} \right) \times 87.6}}{{{\text{a}}.{\text{d}}.{\text{t}}}}$$

2 $${\text{PE}} = { }\frac{{{\text{W}}0 - {\text{W}}1{ }}}{{{\text{W}}0}}{\text{\% }}$$

*∆W* is known as the weight loss per unit area per unit time (g cm^−2^ h^−1^). *W*_1_ and *W*_0_ are wt losses by mg for the treated and untreated coated species; respectively, a is the surface area of steel (cm^2^), d is the C.S. density (g/cm^3^) = 7.85 and t is the time of exposure in h. SEM/EDX morphological combination was surveyed for the coated steel coupons after the direct innuendo to the hazardous saline atmosphere to affirm the weight loss method.

### X-ray diffraction (XRD) of the coating layers after the exposure to salt spray aggressive fog

XRD patterns were checked utilizing PANanalytical X'pent PRO MPD X-ray Diffractometer with Cu Kα radiation (λ: 0.15418 nm, 2θ range 5°–80°, scanning mode: continuous, scan step size: 0.04° and scan step time: 0.5 s) to survey the investigated coating layers after the direct exposure to salt spray aggressive fog for 500 h. The coating layer ingredients, including rusting deposits, formed salts, and deteriorated coating materials, were collected using a sharp cutter and perfectly ground to be measured.

### Mechanical and UV aging durability properties

The mechanical durability testing was assessed using cross-hatch cut adhesion according to ASTM D 3359-17^34^, pull-off adhesion using ISO4624:2002^35^, bend utilizing the standard of ASTM D 522-93a^36^ in addition to resistance to abrasion resistance employing the ASTM D 4060-95^37^. UV durability survey (G151-19, ASTM D4587-11, and G154) was performed to evaluate the UV resistance of coatings^38–40^.

## Results and discussion

### Physico-analytical properties and geometry confirmation of ligands and their complexes

Table 1 illustrated the given physico-analytical properties of the investigated MF, Bipy, and their complexes (Zr(IV) and Cu(II)), and demonstrated elementally their chemical formula. Figure 1c and d offered the stoichiometry of complexes which clarified as 1 M^n+^: 1 M.F. 1: Bipy^29,41,42^ and the chelation information. Molar conductance measurements were investigated to detect the anion's location around the coordination sphere. According to the delivered data depicted in Table 1, the effective magnetic moment (μ_eff_) of the Cu(II) complex was found at 1.70 B.M. (paramagnetic) and offered an octahedral geometrical arrangement^29,43^. Also, octahedral structure geometry was displayed for Zr(IV) with diamagnetic characteristics. As depicted in Table 2, UV. Vis. Spectra offered four bands at 373, 360, 310, and 290 nm, which referred to n-π^*^ and π-π^*^ transitions of the free MF ligand^29,44^. The given data affirmed the appearance of two signals at 284 and 347 nm, which is characteristic of π-π^*^ and n-π^*^ transitions of C=C and C=N groups of free Bipy ligand, respectively^29,45,46^. The shift occurred in bands to lower and higher values, and the appearance of new signals for the employed complexes was attributed to the metal ions chelation with ligands^45,46^. Furthermore, Table 2 showed that the Cu(II) had two signals at 540 and 620 nm, attributed to magnetic properties with a distorted octahedral geometry^29,47–49^.

### Models and structural parameters

#### Molecular orbitals (MOs) and frontier

**Figure 3:**
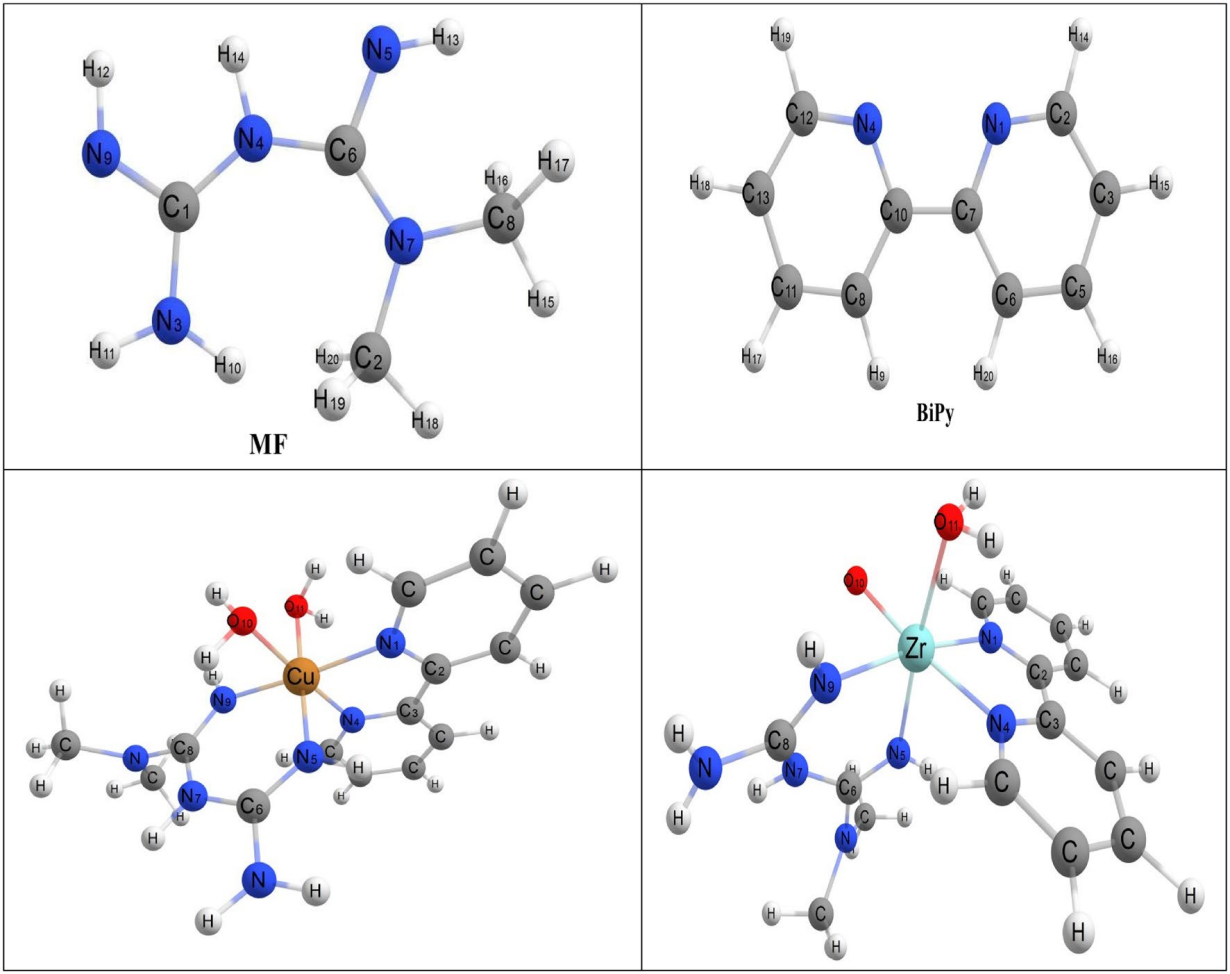
DFT-optimized geometry of MF, Bipy and their metal complexes.

DFT calculations were studied to clarify the structural geometry of the employed ligands and their mixed complexes, as shown in Fig. 3^29^. Based on the electronic system basis, the reactivity of the investigated compounds depends on the HOMO–LUMO energy gap (ΔE), in which a lower ΔE represents high chemical reactivity, and a high ΔE value gives an idea about low reactivity (high stability)^29,50,51^. As depicted in Table 3, the measured ΔE values for the checked compounds affirmed that the complexes had lower ∆E values measured at 0.190 than free ligands (0.365 and 0.280 au) which indicated that mixed complex offered more reactivity via the dispersion in the coating medium. This performance could be attributed to facilitating the electronic transition between the orbitals of these complexes. In addition, there was a relation between the softening and hardening of molecules and their reactivity in which the measured η of Zr(IV) and Cu(II) affirmed their softening properties (η = 0.095 au) and high reactivity than that for free ligands (η = 0.183 for MF and 0.140 for Bipy). According to the depicted data in Table 3, some quantum parameters were measured on the calculated LUMO and HOMO energy estimations. The illustrated electronegativity values (χ = − (H − L/2) of the investigated free ligands and their complexes demonstrated that the complexes had elevated electronegativity values than the free ligands at − 0.294 and − 0.292 counts, respectively. Furthermore, the complexes' electron affinity (A = − L) data showed enhanced values at − 0.199 and − 197. These values indicated that these complexes had more crosslinking affinities with other compounds than free ligands.

#### Excited states

The Time-dependent density functional response theory (TD-DFT) using the G03W program at the B3LYP accurately characterized the UV–Vis. spectra^52–54^. TD-DFT was applied to various molecules and atoms and recently re-subedited to compute separated oscillator strengths and transition energies^55,56^. Bauernschmitt and Ahlrichs^57^ inserted some suggested hybrid functionals in the calculation of excitation energies. These hybrid procedures typically comprised a significant perfection over conventional Hartree–Fock (HF) based procedures. In the present investigation, the optimized geometry was measured and utilized in all subsidiary calculations, and the analysis of wave functions of SCF-MOs was explicitly made. The fraction of various fragments of the complex was suggested by calculating wave functions of the different and participating in the full-wave functions of various states. According to the obtained results, it was found that there was an extension of electron delocalization through the various molecular orbitals.

Mixed n → π* and π → π* transitions represented the defined description of occurring electronic transitions. The obtained HOMO and LUMO energies for MF and Bipy and all checked complexes were listed in Table 4. The HOMO could perform as an electron donor and the LUMO as the electron acceptor in the reaction profile. The composition of the frontier molecular orbital for the studied complexes was given in Table 5. The electron density of HOMO in the case of Cu(II) and Zr(IV) complexes was localized over all atoms of the MF molecule with 98% and 95.4%, respectively, with small portions on the residue of the complex. In the case of LUMO, the electron density of Zr(IV) localized mainly on Bipy with 94.5%^58^. For the Cu(II) complex, the electron density of LUMO was localized with a high percentage of metal ions at 72.3%, and the remaining rate spread over the remaining atoms of the complex. So, the electronic transitions could be described as mixed n → π* and π → π* transitions with a small portion on the Bipy in some complexes and on MF for others, which gave rise to the possibility of MLCT from metal ion to either Bipy or MF (π*).

**Table 4:** Computed excitation energies (au), electronic transition configurations and wave lengths (nm) of the free ligand (MF) and their all obtained stable complexes by using B3LYP/Cep-31G for the, (f ≥ 0.001) f = oscillator strengths.

Complex	E (eV)	λ (nm)	Major contributions	λ (nm), Exp	
MF	4.377 4.039 3.499 3.369	283 306 354 369	H − 1 → L (100%), H → L + 1 (100%), H − 4 → L (100%), H − 1 → L + 2 (50%) H − 1 → L + 1 (50%), H → L (100%) H − 3 → L (50%), H − 3 → L + 1 (50%), H − 2 → L (50%), H − 2 → L + 1 (100%) H − 3 → L (50%), H − 3 → L + 1 (50%), H − 2 → L (50%)	290 310 360 373	π → π* n → π*
Cu(II)	4.3878 3.9595 3.7732 3.429 3.382 3.253 2.311 2.276 2.105	282.57 313.13 328 361.56 366.56 381.17 536.53 544.69 589.05	H − 42 → L (100%), H − 42 → L + 1 (100%), H − 42 → L + 2(100%), H − 1 → L(33.3%) H → L(50%), H → L + 1(33.3%), H → L + 2(25%), H → L + 3(50%), H → L + 4 (25%) H → L + 1 (50%), H → L + 2(33.3%), H → L + 4 (25%), H → L + 3(25%) H → L + 1 (33.3%), H → L + 2 (25%), H → L + 3 (50%), H → L + 4(25%). H − 40 → L + 2 (100%), H − 40 → L + 3 (100%), H − 1 → L + 3(25%), H → L + 2 (25%), H → L + 4(25%) H − 1 → L + 1(33.3%), H − 1 → L + 2 (25%), H − 1 → L + 3 (25%) H − 1 → L (33.3%), H − 1 → L + 1 (33.3%), H − 1 → L + 2 (25%), H − 1 → L + 3 (25%) H − 1 → L + 1 (33.3%), H − 1 → L + 2 (25%) H − 1 → L (33.3%), H − 1 → L + 2 (25%), H − 1 → L + 3 (25%)	290 320 370 540 620	π → π* n → π* LMCT d → d
Zr(IV)	4.2138 3.444 3.1492 2.7385 2.516	294.23 359.96 393.69 452.53 492.8	H → L (50%), H → L + 1 (50%), H → L + 2 (33.3%), H → L + 3 (50%), H → L + 4 (33.3%), H → L + 5 (33.3%), H → L + 7 (33.3%), H − 2 → L (50%), H − 2 → L + 2 (100%), H − 2 → L + 3 (50%), H − 2 → L + 4 (50%), H − 2 → L + 7 (50%) H − 2 → L (50%), H − 2 → L + 3 (50%), H − 2 → L + 4 (50%), H − 2 → L + 7 (50%) H − 4 → L (100%), H − 4 → L + 2(100%), H − 4 → L + 3 (100%), H − 4 → L + 4 (100%), H − 4 → L + 5 (100%), H − 4 → L + 7(100%), H − 4 → L + 9 (100%), H − 4 → L + 10 (100%) H − 1 → L (100%), H − 1 → L + 2(50%), H − 1 → L + 4 (50%), H − 1 → L + 5 (50%), H − 1 → L + 7 (100%), H → L + 1(50%), H → L + 2 (33.3%), H → L + 3 (50%), H → L + 4 (33.3%), H → L + 5 (33.3%), H → L + 7 (33.3%) H − 1 → L + 1 (100%), H − 1 → L + 2(50%), H − 1 → L + 3 (50%), H − 1 → L + 4 (50%), H − 1 → L + 5 (50%), H → L (50%), H → L + 2 (33.3%), H → L + 4 (33.3%), H → L + 5 (33.3%), H → L + 7 (33.3%)	295 320 370 532	π → π* n → π* LMCT

**Table 5:** Composition of the frontier molecular orbital for all studied complexes by using DFT/B3LYP/Cep-31G.

Fragments	H − 3 (%)	H − 2 (%)	H − 1 (%)	H (%)	L (%)	L + 1 (%)	L + 2 (%)	L + 3 (%)
Cu(II)	1.4	0	1.5	1.5	72.3	7.4	29.9	72
L1	95.4	1.8	1.8	98	9	1.2	2.6	6.3
L2	3.1	98.2	95.8	0	9.8	90.7	65.2	14
Oxygen	0.1	0	0.9	0.5	8.9	0.7	2.3	7.7
Zr(IV)	1.3	0	1.9	0	5	11.5	1.5	17.5
L1	91.5	4.3	1.1	95.4	0.5	17	98.5	54.2
L2	5.1	94.2	13.8	0	94.5	68	0	25.3
Oxygen	2.1	1.5	73.2	4.6	0	3.5	0	3

**Figure 4:**
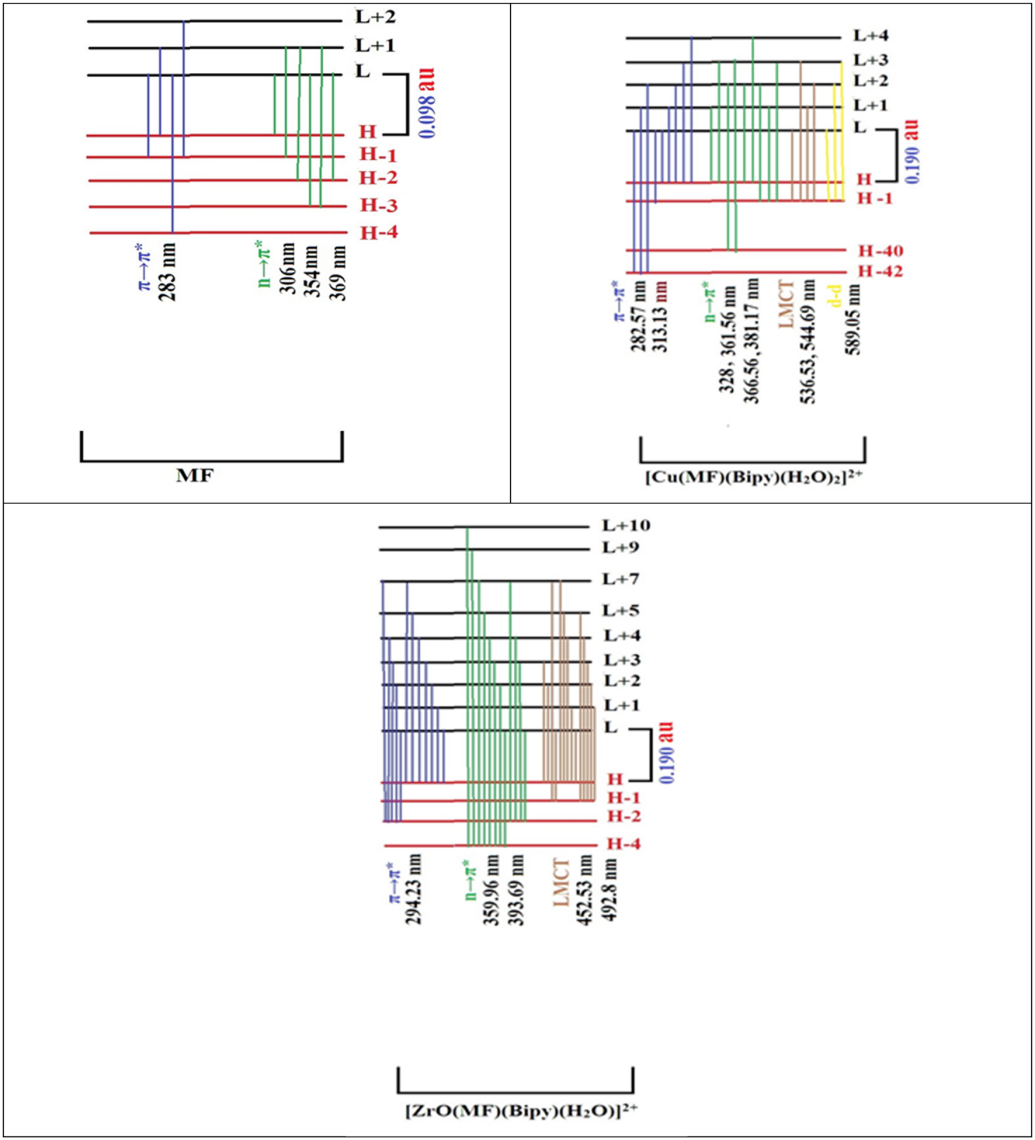
TD-DFT calculated electronic transitions in MF and their metal complexes by using DFT calculations.

Different excited states for each complex were measured. The employed electronic transitions for the studied complexes were displayed in Fig. 4. The electronic transition configurations computed excitation energies (au) and wavelengths (nm) of the checked complexes were illustrated in Table 4. By utilizing theoretical TD-DFT studies, all the complexes exhibited electronic transitions assigned to d-d shifts for Cu(II) at 589.05 nm, and these values agreed with the spectrum of the Cu(II) complex (620 nm). Also, the Cu(II) complex had transition characteristics for the LMCT band at 536.53 and 544.69 nm, and this was typical for the distorted octahedral structure.

In the case of free MF, as shown in Fig. 4, there were four bands, the 1st one at 283 nm, which was assigned to π → π* transition and resulted from four shifts from H, H − 1, and H − 4 with different proportions to L, L + 1, and L + 2. The 2nd, 3rd, and 4th bands at 306, 354, and 369 nm, respectively, and these values assigned to n → π* transition resulted from H, H − 1, H − 2, and H − 3 to L and L + 1with different proportions.

As shown in Fig. 4, the Cu(II) complex exhibited four absorption bands. The 1st one resulted from H − 42 → L (100%), H − 42 → L + 1 (100%), H − 42 → L + 2(100%), and H − 1 → L(33.3%) at 282.57 nm, which were assigned to π → π* transition. The H − 1 orbital was localized on the 2nd ligand with a percent of 95.8%, mainly on nitrogen atoms of the Bipy ligand^58^. The 2nd excited state at 313.13 nm was resulted from H → L (50%), H → L + 1(33.3%), H → L + 2 (25%), H → L + 3(50%), H → L + 4 (25%), whereas the H was localized on 1st ligand molecule with the percent of 98%, especially on the two nitrogen atoms of MF. The L was localized on Cu(II) ion with 72.3%, L + 1 was localized on Bipy with 90.7%, L + 2 was localized on Bipy^58^, and Cu(II) ion with 65.2% and 29.9%, respectively, and L + 3 was localized on Cu(II) ion with 72%. These two excited states were equivalent to the experimental band at 290 nm, appointed to π → π* transition. The 2nd absorption band at 320 and 370 nm, which was established to n → π* transition, resulted from four theoretical bands at 328, 361.65, 366.56, and 371.19 nm. There were four transitions, as given in Table 4. The 3rd absorption band at 536.53 nm which composed of four changes H − 1 → L (33.3%), H − 1 → L + 1 (33.3%), H − 1 → L + 2 (25%), and H − 1 → L + 3 (25%), and at 544.69 nm which composed of two transitions H − 1 → L + 1 (33.3%) and H − 1 → L + 2 (25%), appointed to ligand metal charge transfer (LMCT), and all changes of this band were performed from H − 1 orbital which localized on Bipy with 95.8% mainly on the two nitrogen atoms. This theoretical value was in agreement with the experimental value at 540 nm. The 4th band of this complex appeared at 544.69 nm, which is characteristic of the d-d transition, which is composed of three shifts H − 1 → L (33.3%), H − 1 → L + 2 (25%), and H − 1 → L + 3 (25%). This band was in agreement with the experimental band at 620 nm, as listed in Table 4.

The Zr(IV) complex had three absorption bands. The 1st band was appointed to π → π* transition and resulted from twelve electronic transitions (H → L (50%), H → L + 1 (50%), H → L + 2 (33.3%), H → L + 3 (50%), H → L + 4 (33.3%), H → L + 5 (33.3%), H → L + 7 (33.3%), H − 2 → L (50%), H − 2 → L + 2 (100%), H − 2 → L + 3 (50%), H − 2 → L + 4 (50%), and H − 2 → L + 7 (50%)) at 294.23 nm. This theoretical band was in agreement with experimental band at 295 nm. The 2nd band which assigned to n → π* transition resulted from four electronic transitions (H − 2 → L (50%), H − 2 → L + 3 (50%), H − 2 → L + 4 (50%), and H − 2 → L + 7 (50%)) at 359.96 nm and eight electronic transitions (H − 2 → L (50%), H − 2 → L + 3 (50%), H − 2 → L + 4 (50%), H − 2 → L + 7 (50%)) at 393.69 nm and it was in agreement with the experimental bands at 320 and 370 nm. The 3rd band which characteristic for LMCT resulted from eleven electronic transitions (H − 1 → L (100%), H − 1 → L + 2(50%), H − 1 → L + 4 (50%), H − 1 → L + 5 (50%), H − 1 → L + 7 (100%), H → L + 1(50%), H → L + 2 (33.3%), H → L + 3 (50%), H → L + 4 (33.3%), H → L + 5 (33.3%), and H → L + 7 (33.3%)) at 452.53 nm and ten electronic transitions (H − 1 → L + 1 (100%), H − 1 → L + 2(50%), H − 1 → L + 3 (50%), H − 1 → L + 4 (50%), H − 1 → L + 5 (50%), H → L (50%), H → L + 2 (33.3%), H → L + 4 (33.3%), H → L + 5 (33.3%), and H → L + 7 (33.3%)) at 492.8 nm. These two theoretical bands were in agreement with experimental band at 532 nm as displayed in Fig. 4.

### The protective performance of coatings

#### EIS measurements

**Figure 5:**
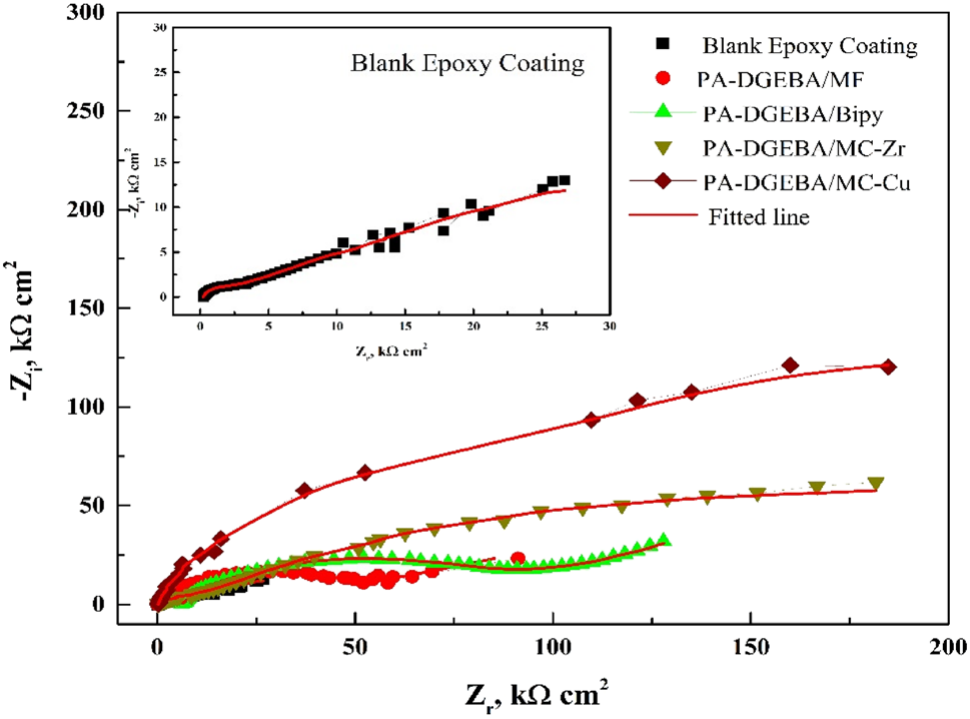
Nyquist plots with fitted lines for different modified epoxy coated steel films against blank conventional epoxy after immersion in oil-wells formation water.

**Figure 6:**
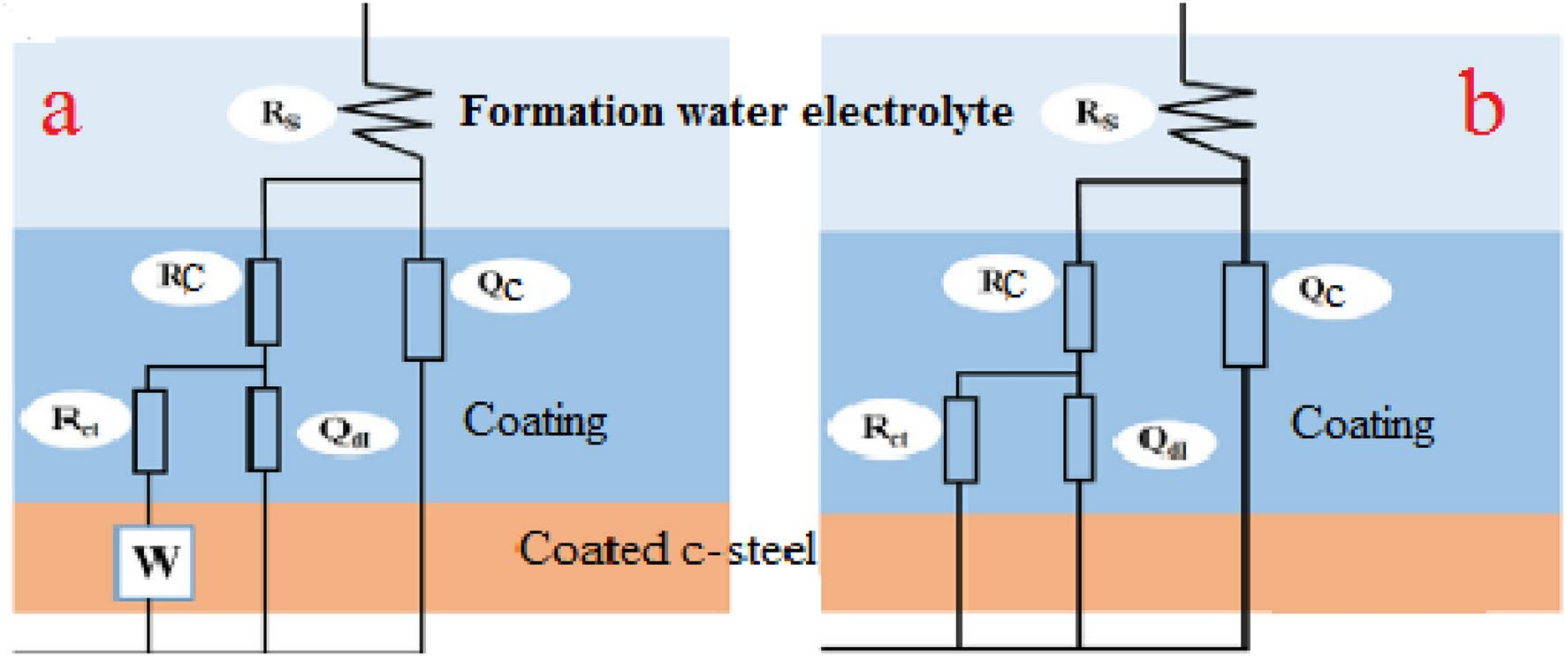
The suggested model for the equivalent circuit used for fitting the impedance parameters of coating systems in oil-wells formation water.

After designing the homogeneous modified coatings against neat epoxy applied on C-steel coupons surface, and obtaining complete curing, the investigated coatings were evaluated to check their protective behavior through EIS and weight loss measurements. In addition, it was found that the free ligands and their complexes could be used as coloring pigment materials with high loading levels via the epoxy binder^59^. Furthermore, the abbreviations of coating codes could be clarified as PA-DGEBA/MF (polyamine cured epoxy of DGEBA-type modified with MF ligand), PA-DGEBA/Bipy (polyamine cured epoxy of DGEBA-type modified with Bipy ligand), PA-DGEBA/MC-Zr (polyamine cured epoxy of DGEBA-type modified with Zr(IV) mixed complex), and PA-DGEBA/MC-Cu (polyamine cured epoxy of DGEBA-type modified with Cu(II) various complex). For affirming the anti-corrosion properties of the checked treated epoxy coated layers, the electrochemical demeanor was discussed using EIS measurements in oil-wells formation water solution (highly corrosive environment). In addition, the corrosive species diffusion via the surface-modified MF and Bipy and their mixed Cu(II) and Zr(IV) coated layers was clarified using this non-destructive analysis. As illustrated in Fig. 5, Nyquist plots affirmed the inhibiting performance of the investigated modified coatings against blank epoxy employing the same concentration (%) by wt. from the modifier compounds (crosslinkers). As illustrated in Fig. 5, the semicircle-shaped curves recorded an increase in the diameter size of the Nyquist curves with the treatment by modifier crosslinkers. They demonstrated the highest diameter for surface-modified Cu(II) epoxy composite coated film. The reinforcement in the intensity of crosslinking due to the excellent pervasion of Cu(II) compounds having paramagnetic properties via the epoxy binder in which enhanced the internal surface interactions, boosted the electronegativity and electron affinity values. Then, the interfacial adhesion was enhanced and supported via the donor (coating)/acceptor (CS) interactions. After that, an increase in the impedance of the surface-modified Cu(II) epoxy coating layer reflected on enhancing the semicircle diameter of Nyquist plots, thereby achieving the best corrosion inhibiting demeanor compared to the other coated steel coupons. By utilizing the equivalent circuits (EECs) to recognize the investigated coatings' inhibiting mechanisms, the impedance fittings were underlined. CPE (The constant phase element) offered the performance of the employed coatings. The electrochemical performance of the various coated layers was demonstrated using EEC with two series–parallel *R*-CPE circuits, as shown in Fig. 6. Figure 6a was employed to analyze the impedance spectra including Warburg impedance as illustrated in case of PA-DGEBA/MF and PA-DGEBA/Bipy coatings due to appearance of pinholes over the coated films and this behavior affirmed the diffusion of formation water electrolyte to the steel surface via these coating pores against full deterioration in case of blank epoxy coating. Figure 6b was applied to the other coating systems (PA-DGEBA/MC-Zr and PA-DGEBA/MC-Cu coatings), in which the coating layer protect the steel surface without presence of surface pores. The electrochemical parameters using the presented fittings were illustrated as *R*_*c*_ (coating pore resistance); *R*_*s*_ (electrolyte or solution resistance); *n*_*1*_ and *n*_*2*_ (the exponent of CPE and *Q*_*dl*_, respectively); *Q*_*c*,_ and *Q*_*dl*_ (magnitude of CPE and the double-layer capacitance, respectively).

Blank epoxy coated layer, as offered in Fig. 5, illustrated the highest dispersal of corrosive species via the pinholes and voids dispersed over the coating surface, and this diffusion of ions diminished with the incorporation of modifier molecules. The presence of free pinholes via the untreated epoxy coating led to a decrease in *R*_*c*_ value. It was achieved at 29 kΩ cm^2^, and the corrosive ions were diffused via the zigzag channels to reach the C-steel substrate via the coating. As illustrated in Table 6, *R*_*c*_ values were measured at 514 and 695 for surface-modified MF and Bipy coatings, respectively. The inhibitive mechanism was enhanced with the Zr(IV) and Cu(II) intercalating through epoxy films due to the established overlapping plates of the highly crosslinked networks, thereby prohibiting the transmission paths (open sites) of corrosive ions over the coating surface. Figure 5 illustrated that PA-DGEBA/MC-Cu coated layer realized the highest *R*_*c*_ value at 940 kΩ cm^2^, indicating the least number of open pores over the coating surface. The exhibited protective behavior of PA-DGEBA/MC-Cu coating was related to the spreading of Cu(II) complex compounds with paramagnetic characteristics, d-d transitions, octahedral geometries through the epoxy film. Cu(II) complex molecules could occupy the free spaces (voids) and reinforce the intensity of crosslinking, thereby reducing the segmental chain motions and boosting the interfacial adhesion. In all events, a diminishing in *Q*_*dl*_ value could be assigned to the filling of interfacial delaminated zones by the modifier compounds^60^. Data illustrated in Table 6 emphasized that PA-DGEBA/MC-Cu coating achieved the least *Q*_*dl*_ estimation at 0.08 µF cm^−2^. In the case of the neat coated layer, the *Q*_*dl*_ value was calculated at 115 assessment and achieved the highest double layer capacitance value (the least inhibition behavior). The corrosion-inhibiting efficiency (*η*) was gauged according to the following equation^29,61,62^:

3 $$\eta\%=\left({\frac{{R_{ct-}R^{O}_{ct}}}{{R_{ct}}}}\right){}\times{}100$$

where *R*^*o*^_*ct*_ and *R*_*ct*_ are the charge transfer resistances of conventional epoxy and treated coating, respectively. Data offered in Table 6 demonstrated that *R*_*ct*_ estimations were increased by increasing the loading of modifier material with high atomic weight and magnetic properties. In addition, *R*_*ct*_ of blank coating fulfilled the most negligible value at 27.5 kΩ cm^2^. Also, PA-DGEBA/MC-Cu displayed the highest *R*_*ct*_ at 940 kΩ cm^2^ and exhibited the best protection efficiency at 97.07%.

**Table 6:** EIS Parameters for different modified epoxy coated films against blank conventional epoxy (70–75 µm thickness) in oil-wells formation water solution.

Sample	*R*_*ct*_ (kΩ cm^2^)	*Q*_*dl*_ (µF cm^−2^)	*R*_*c*_ (kΩ cm^2^)	*n*_*1*_	*n*_*2*_	*Q*_*c*_ (µF cm^−2^)	*R*_*s*_ (kΩ cm^2^)	*η* (%)
Blank epoxy coating	27.5	115	29	0.57	0.61	1.3170	0.265	–
PA-DGEBA/MF	608	19	514	0.73	0.57	0.1900	0.399	95.47
PA-DGEBA/Bipy	681	7	695	0.53	0.87	0.0500	0.461	95.96
PA-DGEBA/MC-Zr	933	0.19	912	0.61	0.65	0.0030	2.798	97.05
PA-DGEBA/MC-Cu	940	0.08	930	0.53	0.51	0.0009	3.007	97.07

#### Weight loss method using salt spray corrosion test (corrosion rate and protection efficiency) using EDX/SEM combination

**Figure 7:**
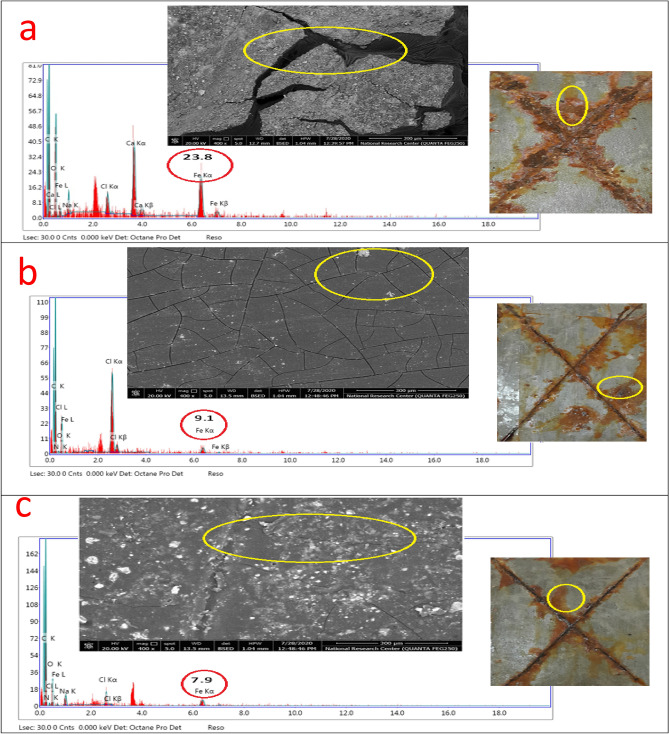

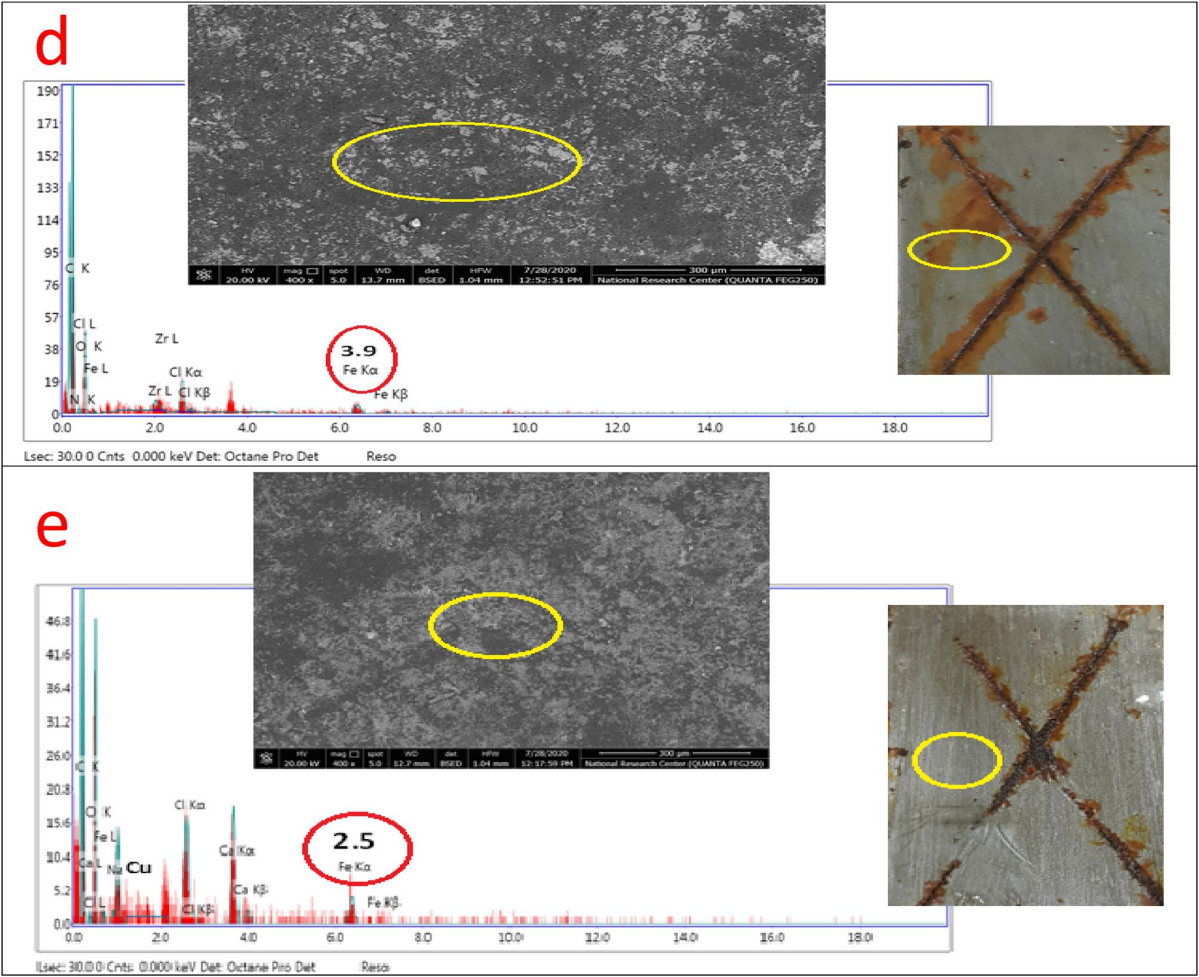
EDX/SEM image collections of the investigated coated films (**a**) Unmodified conventional epoxy, (**b**) PA-DGEBA/MF, (**c**) PA-DGEBA/Bipy, (**d**) PA-DGEBA/MC-Zr and (**e**) PA-DGEBA/MC-Cu after the direct exposure to salt spray fog at severe conditions.

**Figure 8:**
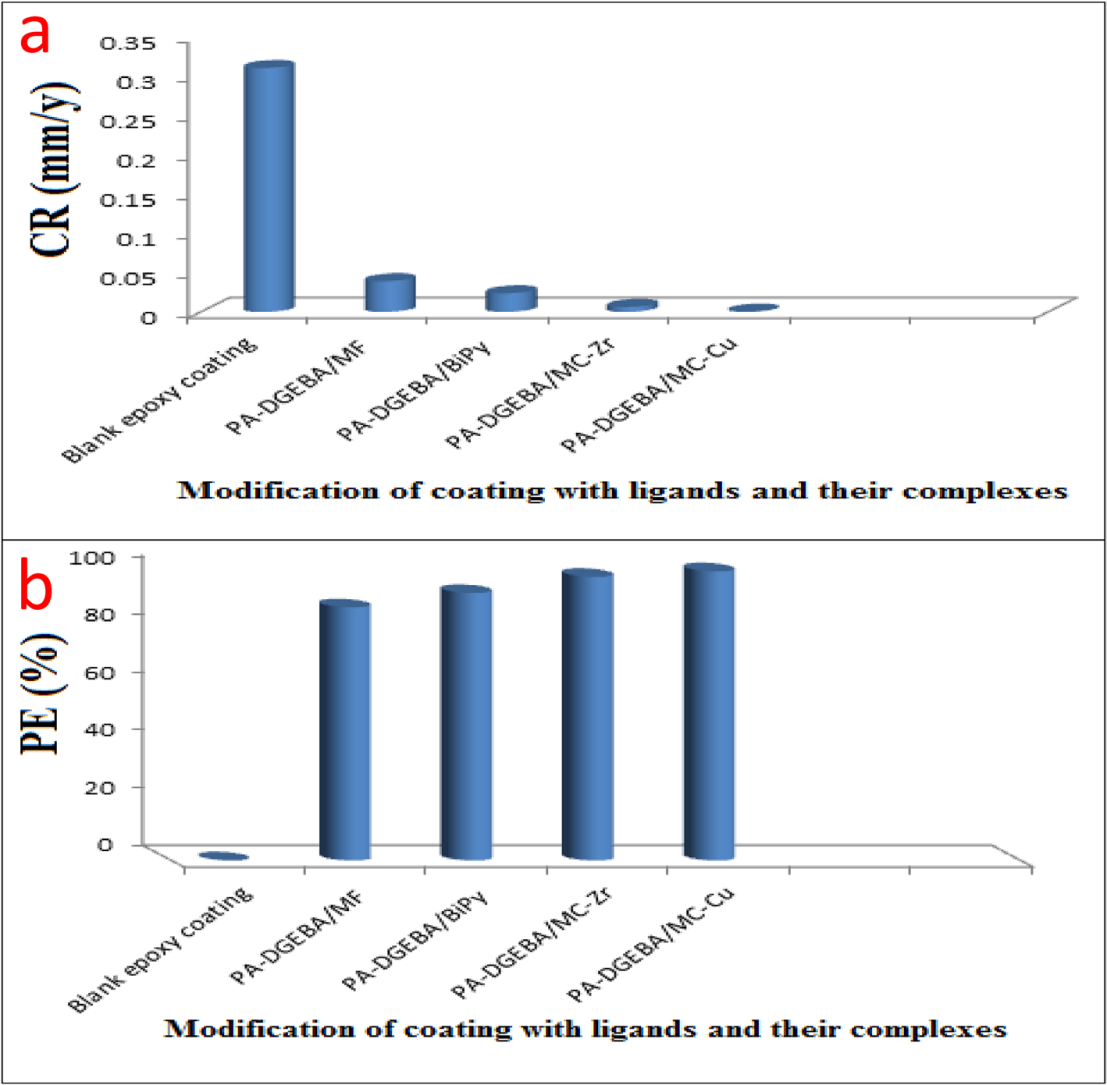
illustrates the protective performance of the investigated coatings (**a**) Corrosion rate (CR) and (**b**) Protection efficiency (PE%) after the direct exposure to salt spray fog for 500 h.

Based on the EDX/SEM image collections and visual inspected photographs of the checked untreated and surface-treated MF, Bipy, Cu(II), and Zr(IV) coatings after the exposure to aggressive salt spray fog as displayed in Fig. 7, the inhibitive behavior performance was studied and confirmed. The modifier moieties were incorporated by the same ratio, and the coatings were applied with the same cured thickness. To support this behavior, the obtained weight loss results for untreated and surface-treated coatings after the innuendo to salt spray corrosive fog (500 h) were observed in Table 7. Conventional (blank) coating manifested the upper corrosion rate (CR) estimation at 0.30964 mm/y, offering the least protection. This behavior was due to the passing of corrosive species (O_2_, Cl^−^, and H_2_O) via the zigzag open zones presented over the coating surface and reaching the CS substrate. The intercalating of the modifier compounds reinforced the PE and decreased the CR values. PA-DGEBA/MF and PA-DGEBA/Bipy coatings presented preferable inhibitive demeanor versus blank one and fulfilled lower CR estimation at 0.03896 and 0.02356 mm/y with improved PE values at 87.41 and 92.38%, respectively. An ascending increase in the corrosion inhibiting performance was noticed for surface-treated Zr(IV) and Cu(II) coatings, respectively, in which the modified epoxy film with Cu(II) attained the least CR rating at 0.00049 mm/y with elevated PE of 99.84%. EDX/SEM image collections, as illustrated in Fig. 7, represented a combination of quantitative and qualitative surface morphological analysis of the investigated coatings^29^. These image combinations could describe the inhibitive performance by measuring the following: (i) The intensity of Fe peak intensity detected by arrows in which high peak count demonstrated weak protective film, and the contrast was correct. (ii) Illustrating the chemical composition of the coating layer with and without the modification by ligands and their complexes and some aggressive elements presented owing to innuendo with corrosive salt spray fog. (iii) Displaying the nitrogen yield due to the coating treatment process by the checked modifier molecules, rust formed as Fe(OH)_3_ molecules and NaOH moieties formed and caused the delamination of the coating surface. Figure 7a demonstrated some excessive corrosion products with big cracks monitored over the coating surface in the case of blank unmodified epoxy. By surveying Fig. 7a, the coating layer achieved the highest Fe account (the least protection), indicating intensive damage represented in the shape of rusting and coating delamination. By loading the coating layer with ligands and their complex compounds (MF, Bipy, Zr(IV), and Cu(II)), respectively, an enhancement in the corrosion inhibiting performance and a diminishing in the rusting, iron peak count, and delamination grade as illustrated in Fig. 7b–d. The coated layer's superior inhibitive performance of surface-modified Cu(II) without dispersion of micro-voids and cracks over the coating surface was clearly illustrated in Fig. 7e^29^. The established inter-crosslinking networks via organic/inorganic (DGEBA/Cu(II)) connections were responsible for the superb protective behavior and achieved a minimal iron count without rusting^63^. Furthermore, the modification process by Cu(II) and Zr (IV) complexes were confirmed by the appearance of N, Cu, and Zr atoms in the structure of coating films in addition to the absence of corrosive species^64,65^ as illustrated in Fig. 7d and e. The epoxy coating displayed the decrease in CR by incorporating MF, Bipy, Zr(IV), and Cu(II) molecules, respectively, through the epoxy coating as displayed in Fig. 8a. In addition, the enhancement in PE with the modification process to affirm the protective demeanor was offered in Fig. 8b.

**Table 7:** Weight loss data of conventional unmodified epoxy and modified MF, Bipy, Zr(IV), and Cu(II) epoxy coated films after 500 h exposure in salt spray cabinet using 5% NaCl solution.

Coating code	Initial mass before exposure (mg)	Final mass after exposure (mg)	Mass loss (mg)	CR (mm/y)	PE (%)
Blank epoxy coating	32,183.8	31,490.1	693.7	0.30964	–
PA-DGEBA/MF	33,216.6	33,129.3	87.3	0.03896	87.41
PA-DGEBA/BiPy	34,394.0	34,341.2	52.8	0.02356	92.38
PA-DGEBA/MC-Zr	33,279.8	33,265.7	14.1	0.00629	97.96
PA-DGEBA/MC-Cu	32,697.4	32,696.3	1.1	0.00049	99.84

*CR* corrosion rate, *PE* protection efficiency.

The corrosion inhibiting effect of surface-modified Bipy and MF coatings than in the case of conventional epoxy (blank) could be observed due to reinforcing the ligand amine moiety/epoxy inter-connecting density by the well dispersion of Bipy and MF molecules via epoxy film. According to the detected data presented in Table 1, the paramagnetic characteristics of the Cu(II) complex (μ_eff_ of 1.70) dispersed through epoxy coating was responsible for its multi-protective behavior. In addition, it was observed that Zr(IV) complex molecules were with diamagnetic characteristics. The consolidated epoxy/steel interfacial adhesion and protective demeanor in the case of modified Cu(II) coating could be assigned to boosting the chemical binding affinity between epoxy and Cu(II) active polar moieties because of the magnetic characteristics of Cu(II), thereby reinforcing the intensity of crosslinking and preventing the transmission of corrosive ions via the zigzag paths of the coated film and established a superior inhibitive barricade^66,67^. Also, the high molecular weight of Zr(IV) compared with that of MF and Bipy molecules caused the enhanced inhibitive performance of the modified Zr(IV) coating than treated by MF and Bipy^68^.

#### XRD patterns for the cured coating layers after the exposure to salt spray corrosive fog

**Figure 9:**
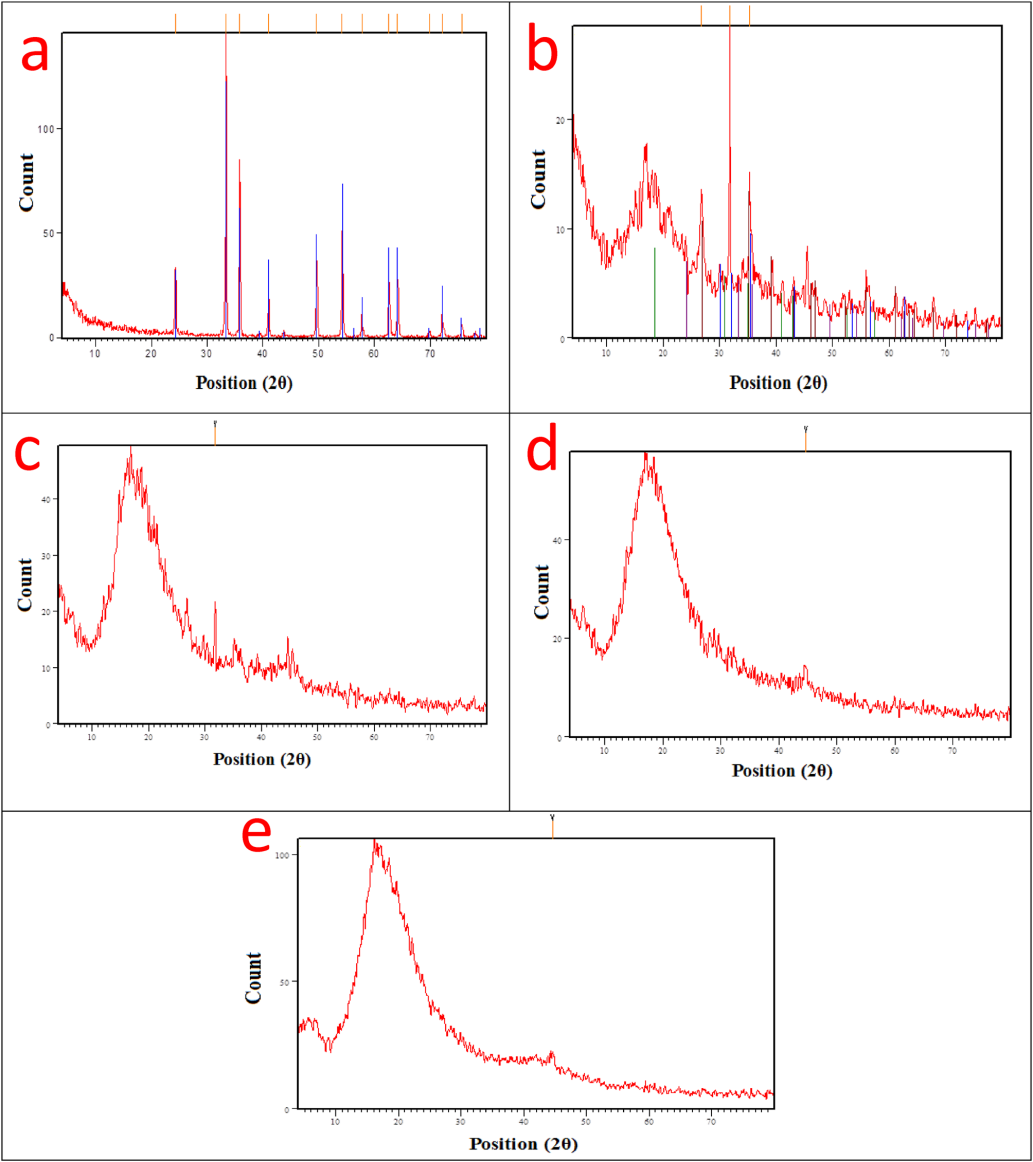
XRD patterns of the checked coating layers (**a**) Unmodified conventional epoxy, (**b**) PA-DGEBA/MF, (**c**) PA-DGEBA/Bipy, (**d**) PA-DGEBA/MC-Zr and (**e**) PA-DGEBA/MC-Cu after the direct exposure to aggressive salt spray fog.

The investigated XRD patterns of the checked coatings layers after performing the salt spray test and exposure to aggressive fog was studied to illustrate the corrosion mitigation performance of the surface-modified coatings against the unmodified blank epoxy. Figure 9a offered the XRD pattern of the top deteriorated cured layer with concentrated rusting of the blank epoxy coating after the exposure to aggressive salt spray fog in which the diffraction peaks appeared at 21, 36, and 53°, corresponding to (110), (111) and (221) planes of iron hydroxide phase (α-FeOOH)^69^. Other diffraction peaks appeared between 35 and 65° in agreement with (311), (400), and (440) planes of the iron oxide phase (γ-Fe_2_O_3_)^69^. The displayed sharp diffraction peaks through the blank coating layer affirmed the appearance of rusting degrees with high intensity, which confirmed the complete deterioration of coating film due to the harmful effect of aggressive salt spray fog. As shown in Fig. 9b, the XRD Pattern of the cured layer of PA-DGEBA/MF coating offered little intensive diffraction peaks between 21 and 65°, which agrees with the appearance of some rusting residues but is lower in the intensity than in the case of blank coating. This behavior indicated the protective action of surface-modified MF epoxy composite coating than in the case of unmodified blank coating. In addition, the amorphous shape that appeared in the XRD pattern, as shown in Fig. 9b, was related to the epoxy coating layer and affirmed the complete crosslinking of MF ligand with the polyamine/epoxy composite. Figure 9c demonstrated the XRD diffraction peaks of PA-DGEBA/Bipy coating with an amorphous shape and fewer peaks between 21 and 65°, which is in agreement with the presence of so few rusting residues than in the case of blank and PA-DGEBA/MF coatings. This performance affirmed the completely crosslinking of Bipy with epoxy and enhanced protective action than in the case of blank and DGEBA/MF coated layers. Figure 9d and e illustrated the diffraction peaks of PA-DGEBA/MC-Zr and PA-DGEBA/MC-Cu coatings, respectively, and offered the absence of any sharp peaks with the ultimate formation of amorphous structures. This behavior asserted the high crosslinking of Cu(II) and Zr(IV) complexes with epoxy, in addition to the absence of rusting and reinforcement in the defensive performance of these coatings^70^.

**Figure 10:**
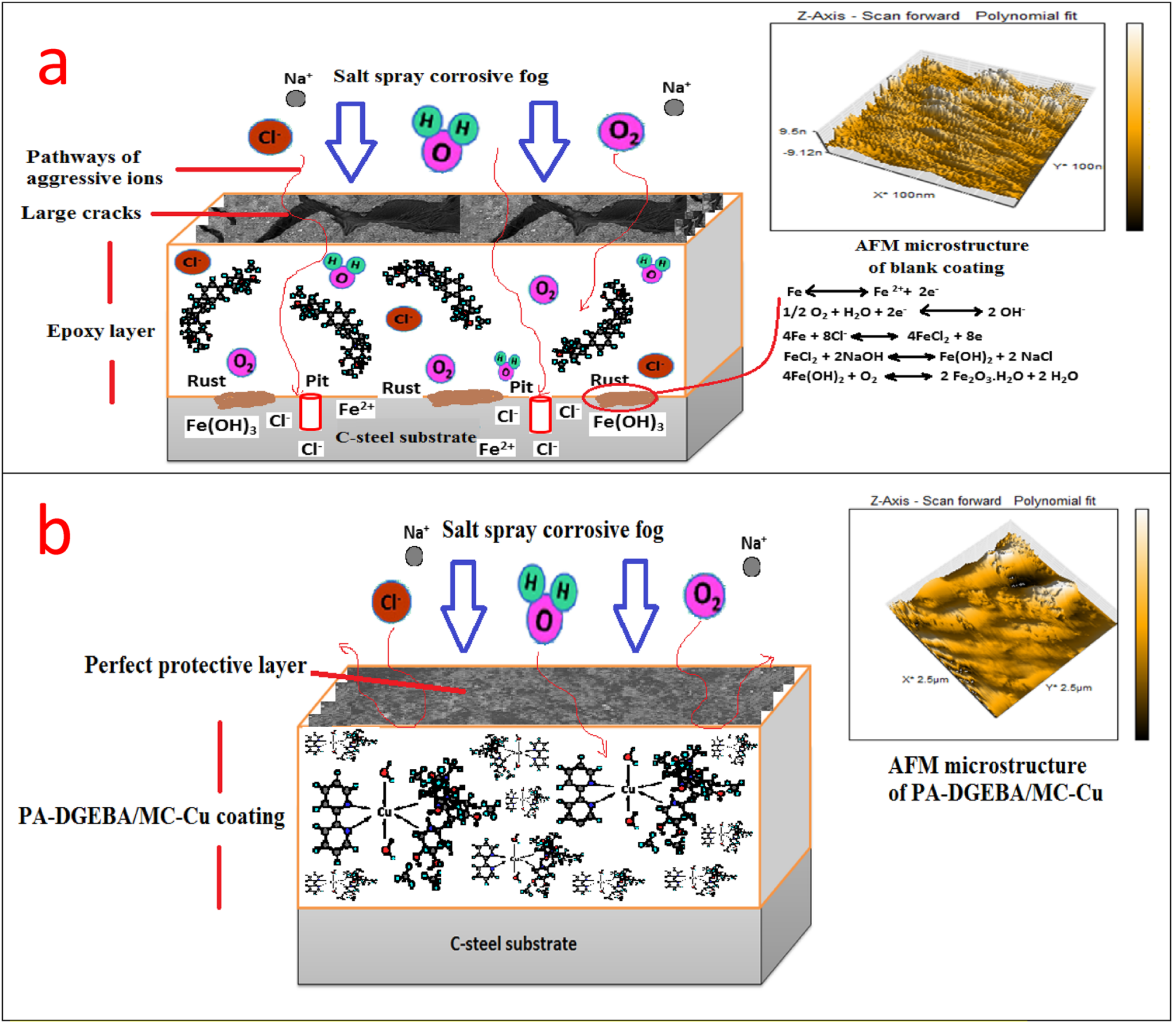
The predicted protective mechanisms of (**a**) Blank unmodified epoxy, and (**b**) PA-DGEBA/MC-Cu coating after the exposure to aggressive salt spray fog.

Based on the chemical composition of the studied cross-linker compounds and their 3D structures, as displayed in Table 8, the synergistic inhibitive effect of the modified cured coatings was offered in Fig. 10, especially the multi-protective mechanism demonstrated by surface-modified Cu(II) coated layer. These protective models could be discussed via the following points; a) The induced physical forces formed between the vacant steel sites, epoxy, and inhibitor moieties in addition to the boosted chemical bonding established between d-vacant orbitals of steel substrate and donating centers (aromatic ring π- electrons, N and O lone pairs and imine (C=N) groups), thereby forming consolidated plastic coating layer^25,65^. So, neat blank epoxy displayed the minimal adherent plastic film as illustrated in Fig. 7a. In addition, blank epoxy coating offered complete film deterioration owing to the dispersion of corrosive moieties (O_2_, H_2_O, and Cl^-^) via the open paths and crackings over the coating surface, leading to the formation of rusting and pitting phenomena^71–74^ as shown in Fig. 10a. With treating the coating layer with inhibitor moieties as MF and Bipy, the inhibitive performance increased and offered few voids and micro-cracks than in the case of neat epoxy because of reinforcing the crosslinking intensity via the acceptor/donor interactions, thereby boosting the chemical bonding, decreasing the number of open channels and resisting the transmittance of corrosive fog through the coated film surface.Then, MF and Bipy enhanced the crosslinking affinity with part of DGEBA, occupied more open surface channels, and consolidated the film stiffness. The higher value of gap energies of free MF and Bipy than in the case of the formed complexes, as depicted in Table 3, diminished the chemical bonding and reinforced the coating/steel interfacial adhesion than in case of modified coatings with the employed complexes. The corrosion inhibiting effect of metal complexes depended effectively on the magnetic moment value, highly atomic weight and electronic transition via the energy levels of these compounds^24^. According to the published research in this area, it was found that the complexes with octahedral geometries and paramagnetic characteristics displayed a reinforced inhibitive behavior than that with square planar geometry and diamagnetism. As shown in Fig. 10b, the surface-modified coating layer with Cu(II) complex demonstrated a superb protective performance. This demeanor was achieved due to the enhanced crosslinking intensity and interface surface interactions resulting from the excellent dispersion of octahedral Cu(II) with a detected magnetic moment value (paramagnetic, μ_eff_ = 1.70), as illustrated in Table 1, and highly atomic weight. These dispersed compounds occupied the free volumes by forming overlapping plates and establishing prohibitive shield preventing the transmission of aggressive species to steel surface via the coating layer. The extra-crosslinking of Cu(II) boosted the inter-coating adhesion, decreasing stress, reducing segmental chain motions, cracking through the coating layer, and preventing the softening in addition to supporting coating stiffness. Furthermore, the measured electronic transitions of Cu(II), as displayed in Fig. 6, illustrated four transitions that reinforced the internal coating/steel interactions with an epoxy vehicle and chemical bonding and decreased the wettability of corrosive solution^24,75,76^. The polynomial fits of 3D micrographs using atomic force spectroscopic measurements for the checked coatings after the direct exposure to the severe fog of the salt spray cabinet were checked for affirmed the preventative performance of the coated film modified by Cu(II) versus the total film deterioration of neat epoxy as displayed in Fig. 10a and b. Blank epoxy, as illustrated in Fig. 7a, demonstrated ultra- some lumps, lamps, cracks, roughened surface, and deteriorated features in the coating film^6,77^. The surface-modified Cu(II) coating layer demonstrated a complex matrix with a smooth multi-protective surface, as illustrated in Fig. 7e.

**Table 8:**
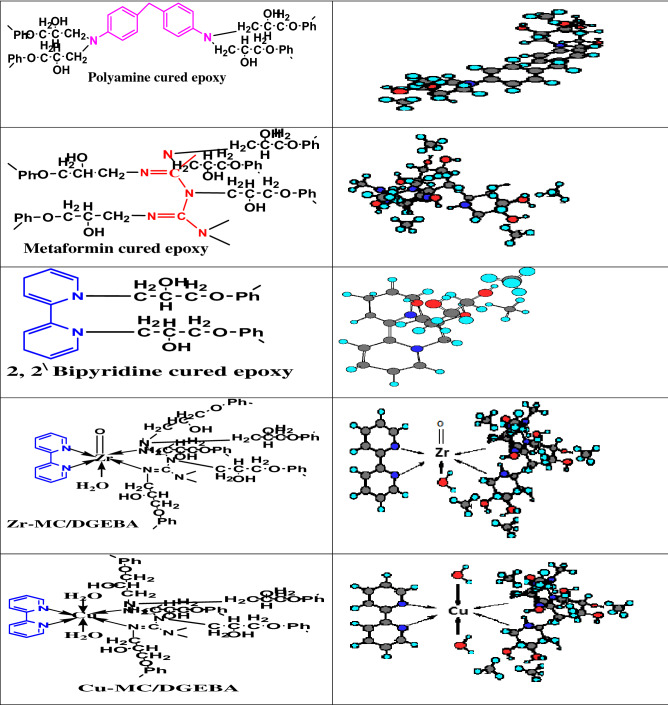
Chemical composition of reactive compounds and their 3D models.

### Mechanical behavior

**Figure 11:**
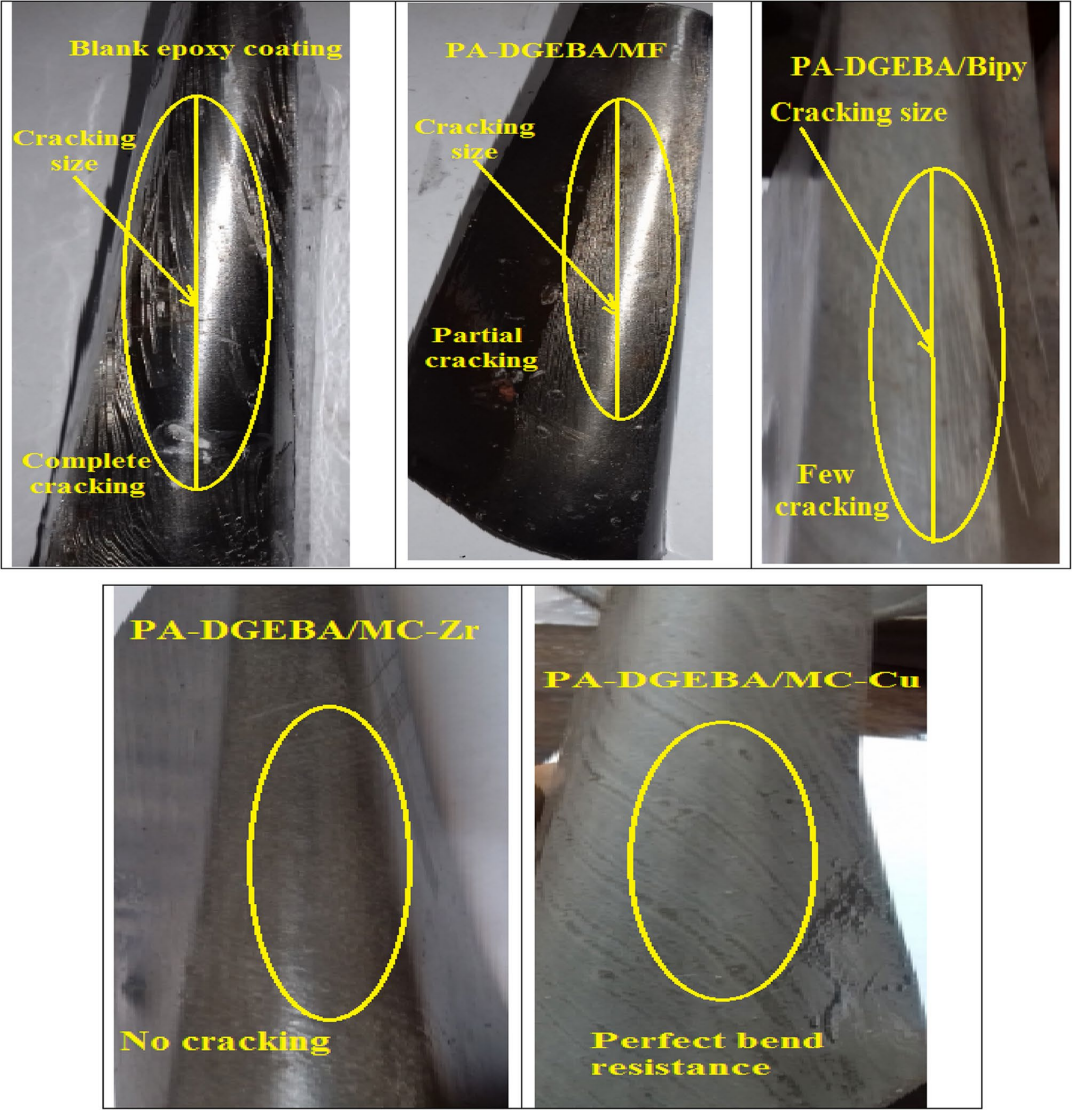
Mechanical bend photographic images for the investigated coated films.

**Figure 12:**
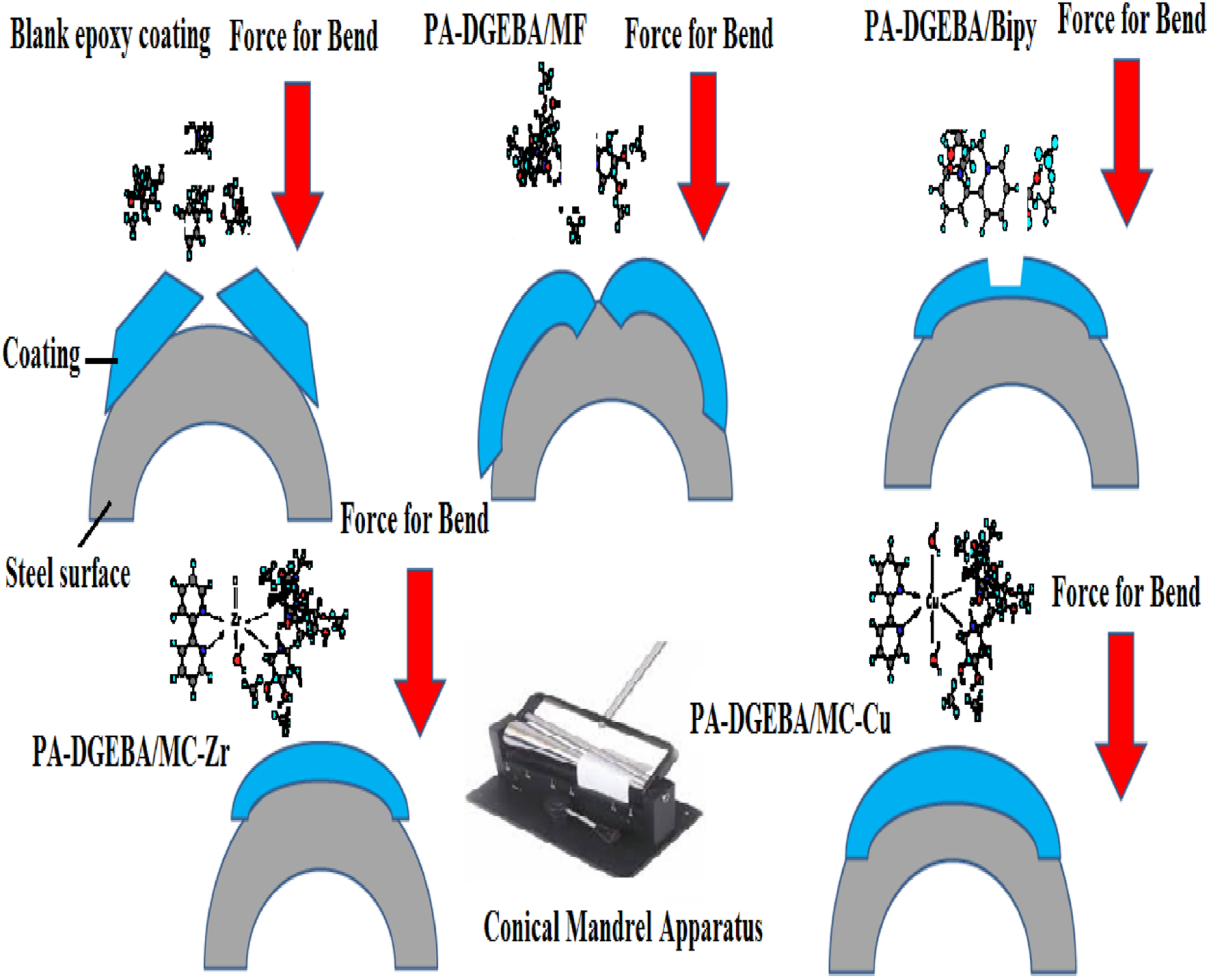
Sketch illustrates the expected behavior of the investigated coated steel samples after applying the bend force.

**Figure 13:**
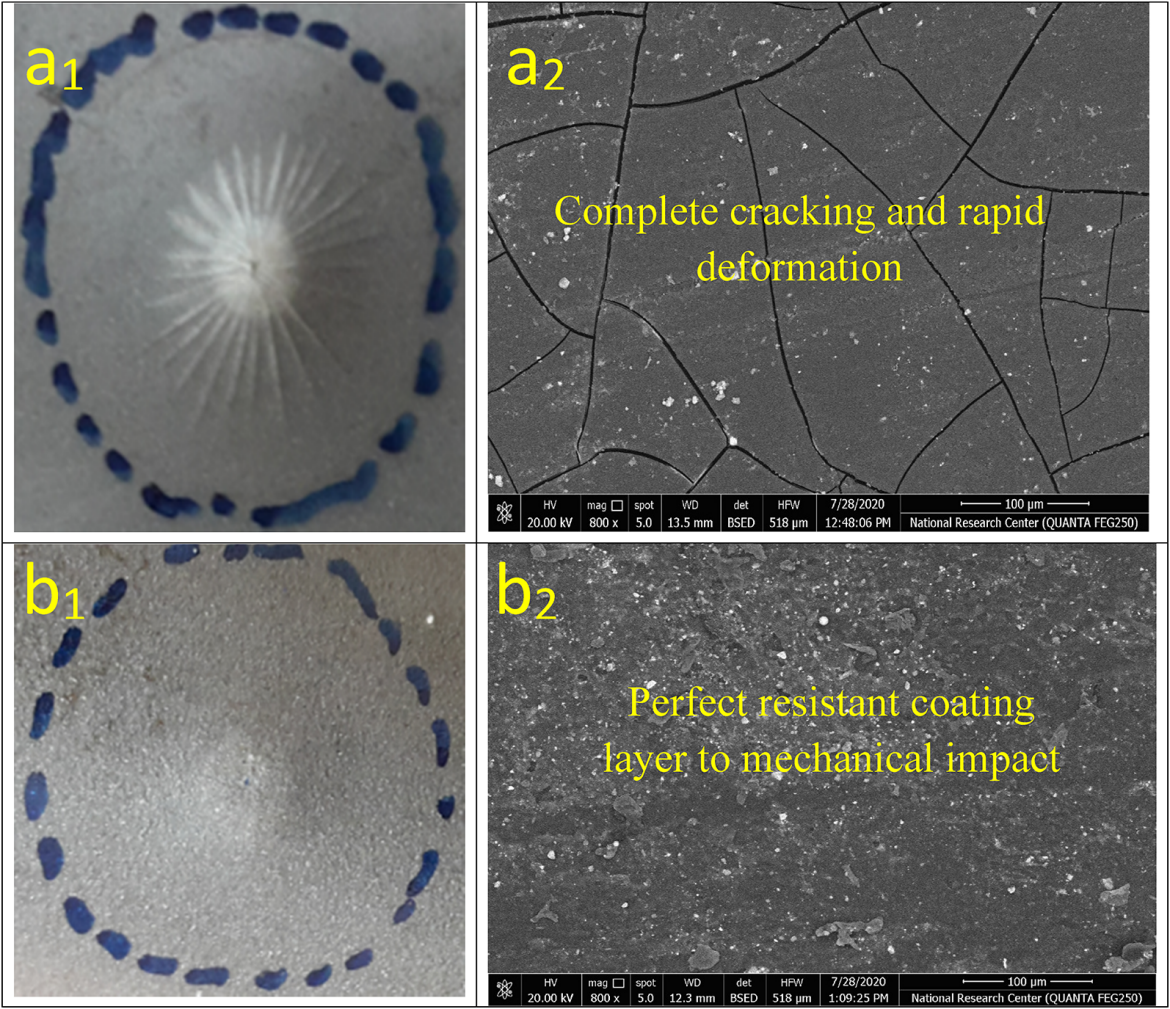
Impact (resistance to rapid deformation) test for the investigated coatings (**a**_1_) Photographic image of blank epoxy, (**a**_2_) SEM image of blank epoxy, (**b**_1_) Photographic image of PA-DGEBA/MC-Cu coating and (**b**_2_) SEM image of PA-DGEBA/MC-Cu coating.

The mechanical tests evaluated the effective mechanical durability performance of the modified coating layers versus total film deterioration in the case of neat epoxy after exposure to the complicated mechanical effort was reviewed by the employed tests as depicted in Table 9. According to the measured data of total elongation (bend property), neat epoxy (unmodified) fulfilled the minimal value at 13. The loading of investigated inhibitor compounds (MF, Bipy, Zr(IV), and Cu(II)) by the same weight ratio improved the bending property. It achieved the maximum elongation for surface-modified Cu(II) coating film at 77. By intercalating with crosslinker modifiers, the pull-off and cross-cut adhesions were enhanced, in which achieved 2.9 MPa and 4B values, respectively, for blank epoxy against consolidated adhesions were fulfilled at 6.3 MPa and 5B, respectively for epoxy coating modified by Cu(II) complex. Also, direct impact (Rapid deformation) resistance was measured and fulfilled the minimal value at 19 J for blank coating. It achieved the highest value for the modified Cu(II) coated film at 59.7 J. Furthermore, abrasion resistance was increased with the treatment by the free and complex compounds, which fulfilled the most negligible mass loss in the coating layer after using the CS-17 Wheel for the surface-modified Cu(II) coating at 11 mg. As illustrated in Table 9, there was a reinforcement in the epoxy coated film stiffness with the epoxy loading by MF, Bipy, and Zr(IV) inhibitor materials. Support in chemical bonding was caused owing to the presence of pyridine molecules with the conjugated aromatic system and nitrogen lone pair of electrons, which enhanced the stability of the coating layer^78^. The durability of presented coatings to bend (flexibility) character was illustrated in Fig. 11. As offered in Fig. 11, the blank epoxy coated panel showed complete cracking with the largest cracking size after the exposure to bending external force. By intercalating epoxy vehicle with the crosslinker modifiers, the investigated modified coated films presented partial and few cracks with smaller cracking sizes than in the case of conventional epoxy. The perfect bend flexibility resistances were offered in the case of modified Zr(IV) and Cu(II) composite coatings. The discussed performance approved the bending durability with the illustrated mechanism proposed in Fig. 12. The distinguished resistance to mechanical efforts by surface-modified Cu(II) and Zr(IV) could be attributed to some fundamental factors as follows; (i) The Paramagnetism of Cu(II) complex (μ_eff_ = 1.70) with four electronic transitions (d → d, π → π*, LMCT and n → π*) in which enhancing the interface interactions and binding affinity via donor/acceptor interface (coating/steel interface). (ii) Cu(II) complex achieved an elevated electronegativity value at − 0.294, electron affinity at − 0.199, and a distorted octahedral geometry. (iii) Well dispersion of complex moieties with highly atomic weights occupied the free zones by the bridging effect with epoxy matrix, diminishing segmental motions and consolidating the film stiffness^79,80^. The rapid deformation due to impact force was offered in Fig. 13a1 and SEM morphology in Fig. 13a2. SEM micrograph of the entire blank film demonstrated several dispersed surface cracks due to the impact effort^81^. Furthermore, the enhanced electron affinity (A) of the Cu(II) complex at -0.199, as illustrated in Table 3, participated in the reinforcement of mechanical resistance of the coating layer. Photographic image and SEM micrograph of modified Cu(II) coated panel displayed a perfect surface free from cracks due to the impact force as illustrated in Fig. 13b1 and b2.

**Table 9:** Mechanical performance properties of conventional unmodified epoxy and modified MF, Bipy, Zr(IV), and Cu(II) epoxy coated films.

Coating code	Bend (total elongation)	Pull-off adhesion (MPa)	Adhesion cross-cut	Direct impact (J)	Abrasion resistance (weight loss(mg)/500cycle/CS-17 wheel/1000gm, wt)
Blank epoxy coating	13	2.9	4B	19	89
PA-DGEBA/MF	25	3.6	5B	31	73
PA-DGEBA/BiPy	28	3.9	5B	45.7	57
PA-DGEBA/MC-Zr	47	4.3	5B	49.5	39
PA-DGEBA/MC-Cu	77	6.3	5B	59.7	11

### UV immovability

**Figure 14:**
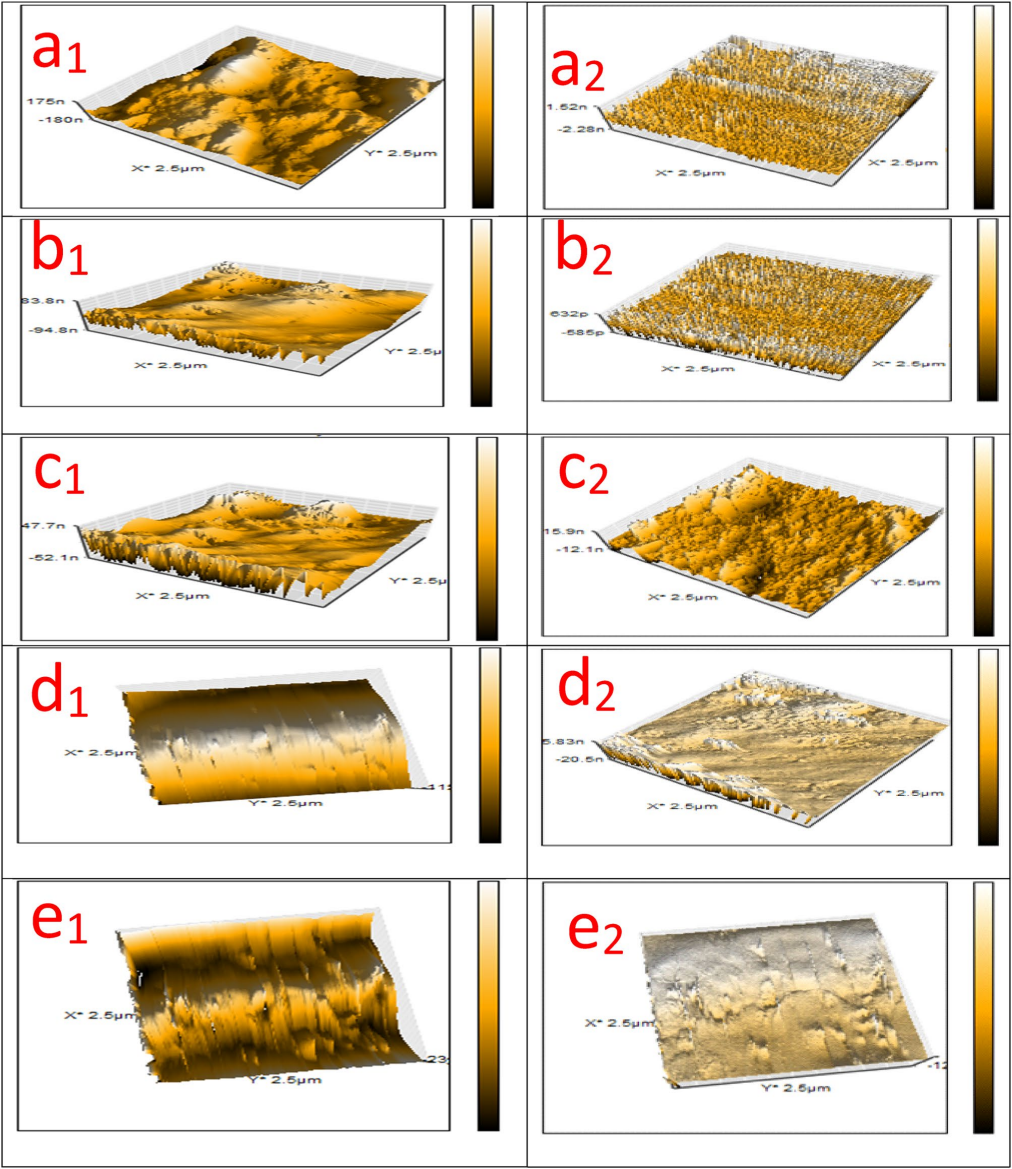
AFM diagrams of the investigated coated films (**a**_1_, **a**_2_) Unmodified conventional epoxy, (**b**_1_, **b**_2_) PA-DGEBA/MF, (**c**_1_, **c**_2_) PA-DGEBA/Bipy, (**d**_1_, **d**_2_) PA-DGEBA/MC-Zr and (**e**_1_, **e**_2_) PA-DGEBA/MC-Cu before and after the direct exposure to solar box UV irradiance for 80 h.

**Figure 15:**
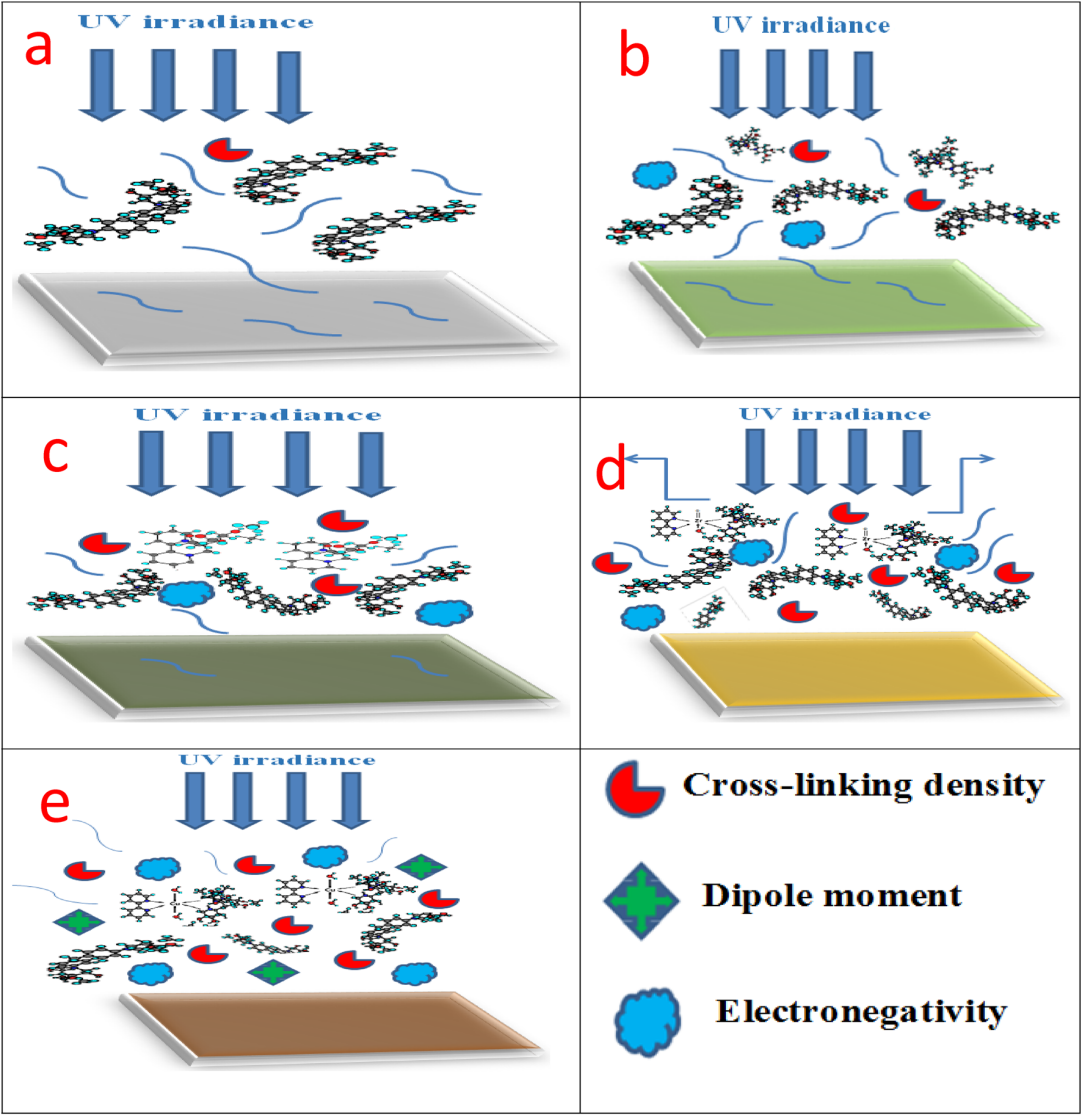
The expected UV resistance mechanisms of the investigated coated films (**a**) Unmodified conventional epoxy, (**b**) PA-DGEBA/MF, (**c**) PA-DGEBA/Bipy, (**d**) PA-DGEBA/MC-Zr and (**e**) PA-DGEBA/MC-Cu after the direct exposure to solar box UV irradiance for 80 h.

For an 80 h period of innuendo at irradiance of 10 and 340 nm wavelength, the UV resistance test was proceeded to measure the ability of the investigated treated coated layers to resist the influence of UV radiation. AFM microstructure survey was offered before and after the exposure to the UV irradiance, as displayed in Fig. 14 and provided in Table 10. Figs. 14a1–e1, illustrated the AFM diagrams of the investigated coatings before the exposure to UV irradiation as perfect applied layers without deterioration in the coated films, but the modifier compounds worked as surface active agents in which decreased the stress and lamps via the coating surface against roughened surface with some lamps in case of blank epoxy. After performing the UV irradiation exposure, conventional coating film showed the lowest resistance to UV irradiation in which blistering was measured and achieved #6 with Dense frequency, adhesion loss (3B), and glossy loss at 20 ^o^ variation from 23.6° to 10.5° due to the UV penetration via the open channels and the ray absorption made by the neat epoxy coating. Figure 14a2 demonstrated disintegration features and lumps that appeared on the coating surface with high roughness due to the hazardous effect of UV irradiance. According to Fig. 14b2, c2, d2, and e2, in the case of the modification process by MF, Bipy, Zr(IV), and Cu(II) compounds, there was an ascending increase and improvement in the UV resistance properties and demonstrated the outstanding performance for the modified Cu(II) coated specimen. According to the results illustrated in Table 10, treated Cu(II) coated film offered the superb UV immovability and fulfilled the maximum behavior at the size of blistering of #8 with few frequencies, 5B adhesion, and glossy variation at 20° (15.42°–14.58°). The suggested UV resistance mechanisms illustrated by the investigated coated films were shown in Fig. 15 and depended on three primary varieties (a) Crosslinking density, (b) Dipole moment value, and (c) Electronegativity. As offered in Fig. 15, the increase in the intensity and the value of these varieties led to an enhancement in UV resistance of the coating layer. So, Blank epoxy coating film demonstrated minimal UV immovability, as shown in Fig. 15a.

**Table 10:** Ultra violet test results of unmodified epoxy and the investigated modified cured epoxy coated panels after exposure to UV irradiance.

Coating code	Blistering		Glossy at 20 variation	Adhesion
	Size	Frequency		
Blank epoxy coating	#6	Dense	23.6°–10.5°	3B
PA-DGEBA/MF	#6	Few	21.13°–15.64°	5B
PA-DGEBA/Bipy	#8	Dense	20.71°–17.77°	5B
PA-DGEBA/MC-Zr	#8	Medium	15.93°–13.11°	5B
PA-DGEBA/MC-Cu	#8	Few	15.42°–14.58°	5B

**Figure 16:**
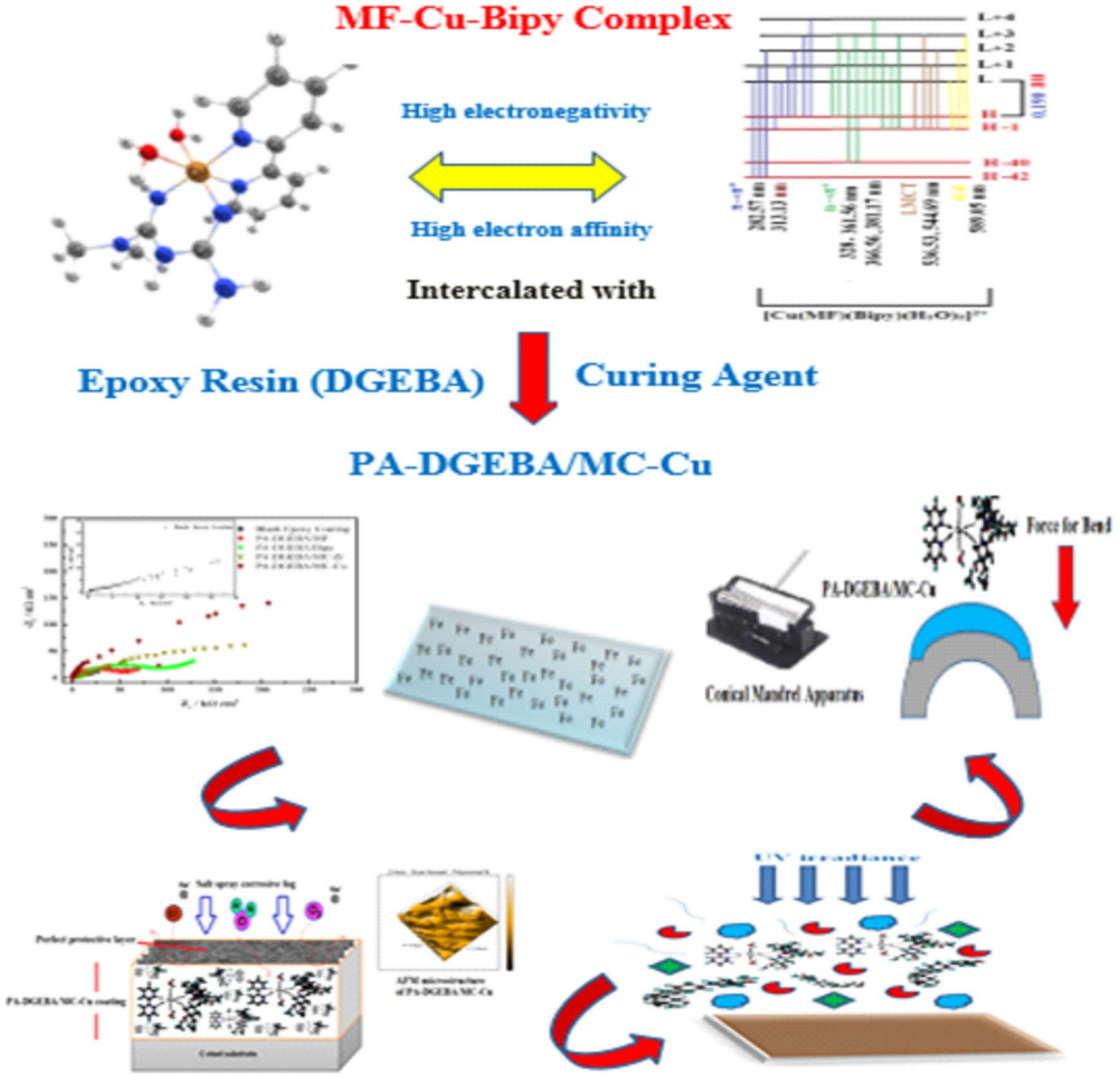
illustrates graphically the multi-functional resistance properties of the investigated surface modified Cu (II) epoxy hybrid composite coating.

Furthermore, there was an increase in the UV resistance property by intercalating epoxy with MF and Bipy via the coating layer, as displayed in Fig. 15b and c, respectively. The outstanding UV immovability results offered by PA-DGEBA/MC-Cu coated specimen as provided in Fig. 15e were assigned to the diffusion of Cu(II) compounds with paramagnetic properties and octahedral geometries and had d-d transitions through the epoxy vehicle. This behavior supported the occupation of open surface sites, which caused a bridging effect with DGEBA and diminished the free spaces through the coating film. Then, the intensity of crosslinking was reinforced, segmental chain motions were reduced, and consolidated stiffness. This demeanor prevented the transmittance of UV irradiation via the coating layer bridges and its absorption and conversion into heat, thereby liberating it to the external atmosphere^29,82^. Figure 15d and e offered the best UV immovability resistance mechanisms by the surface-modified Zr(IV) and Cu(II) coatings, respectively. This behavior depended on the high molecular weight of the Zr(IV) complex with increasing in crosslinking density and electronegativity in addition to the magnetism of the Cu(II) complex with high electronegativity and enhanced crosslinking density. Finally, the multi-functional resistance properties of the investigated epoxy hybrid composite coating encapsulated with MF, Bipy and their mixed Zr(IV), and Cu(II) complexes could be graphically demonstrated as shown in Fig. 16.

## Conclusions

Designed super-protective surface modified epoxy coating formulations were prepared using premium crosslinking agents (MF, Bipy and their Zr(IV), and Cu(II)) to offer multifunctional purposes via evaluating their corrosion mitigation, mechanical and UV durability performances on the C-steel surface. Surface modified Bipy epoxy coating demonstrated an extra-inhibitive layer, proper mechanical and UV immovability properties than modified by MF. Owing to high atomic weight and having octahedral geometry, the treated Zr(IV) coating displayed reinforcement in the durability properties to outdoor effects than for PA-DGEBA/MF and PA-DGEBA/Bipy coated specimens. EIS measurements affirmed the superb protective behavior observed by PA-DGEBA/MC-Cu coating, which achieved the highest *R*_*ct*_ value at 940 kΩ cm^2^, *R*_*c*_ of 930 kΩ cm^2^, the lowest *Q*_*dl*_ at 0.08 µF cm^−2^ and exhibited the highest PE at 97.07%. Based on the weight loss evaluation trial, PA-DGEBA/MC-Cu film attained the minimal CR value at 0.00049 mm/y with a PE of 99.84%. SEM/EDX combination survey of the coated steel films proved the corrosion inhibiting properties of the incorporated ligand and complex molecules. Surface modified Cu(II) coating showed the boosted resistance to mechanical stress in which the film showed pull-off adhesion at 6.3 MPa, cross-cut adhesion of 5B, bend elongation at 77, direct impact resistance at 59.7 J, and abrasion resistance of 11 (weight loss(mg)/500cycle/CS-17 wheel/1000 gm, wt). PA-DGEBA/MC-Cu coated layer illustrated the superb UV irradiance resistance and achieved the maximum behavior at the blistering size of #8 with a few frequency, adhesion class of 5B and glossy loss at 20° from 15.42° to 14.58°. Applications of the investigated modified coatings with Cu(II) and Zr (IV) complexes and their ligands as multifunctional crosslinking agents can be expanded in the future to be utilized on the surfaces of steel tanks and pipelines exposed to the severe aggressive atmospheres, hazardous mechanical efforts, and UV harmful rays.

## Supplementary Information

Supplementary Information.

## Data Availability

All data generated or analyzed during this study are included in this published article and their supplementary materials.
