# Supplementary material for: Robust corrosion guard, mechanical and UV aging properties of metal complex/epoxy hybrid composite coating for C-steel applications
Source: Sci Rep. 2022 Jul 21;12:12483. doi: 10.1038/s41598-022-16348-3 (PMC9304329; doi:10.1038/s41598-022-16348-3)
Supplement: Supplementary file 1 — Supplementary Information. [file 41598_2022_16348_MOESM1_ESM.docx]

| 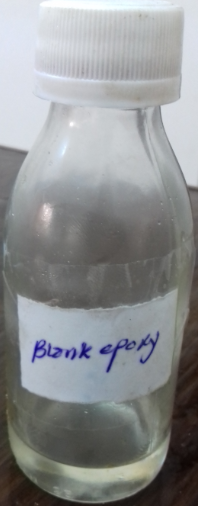 | 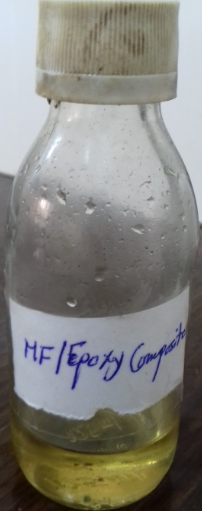 | 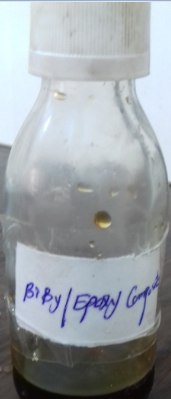 | 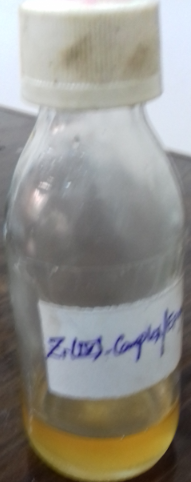 | 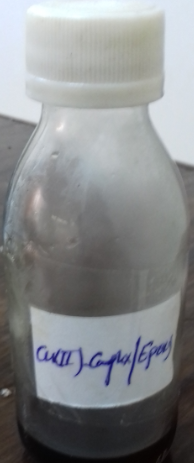 |
| --- | --- | --- | --- | --- |
| **Blank epoxy resin** | **DGEBA/MF lotion** | **DGEBA/Bipy lotion** | **DGEBA/MC-Zr lotion** | **DGEBA/MC-Cu lotion** |

**Fig. S_1_:** The prepared epoxy composite lotions of MF, Bipy ligands and their mixed Zr(IV), and Cu(II)- complexes against blank epoxy.
